# Integrative single-cell RNA-seq and ATAC-seq analysis reveals the key role of inflammatory cell activation in pulmonary arterial hypertension

**DOI:** 10.3389/fimmu.2026.1796116

**Published:** 2026-04-15

**Authors:** Yuanqi Yang, Yang Lei, Boji Wu, Ke Ning, Yang Shen, Xuhong Wang, Jingyu He, Jihang Zhang, Chuan Liu, Zhexue Qin

**Affiliations:** 1 Laboratory of Cardiac Structure and Function at Institute of Cardiovascular Diseases, West China Hospital, Sichuan University, Chengdu, China; 2 Department of Cardiology, West China Hospital, Sichuan University, Chengdu, China; 3 Department of Cardiology, Xinqiao Hospital, Army Medical University, Chongqing, China

**Keywords:** chromatin accessibility, inflammatory cells, pulmonary arterial hypertension, pulmonary vascular remodeling, single-cell sequencing

## Abstract

**Background:**

The cellular heterogeneity and molecular complexity of pulmonary arterial hypertension (PAH) have not been fully elucidated.

**Methods:**

Here, we constructed an integrative transcriptome and chromatin accessibility atlas of PAH mice by using single-cell RNA sequencing and single-cell ATAC sequencing.

**Results:**

In PAH mice, the numbers of granulocytes and monocytes/macrophages in the lung tissues were increased, and the hypoxia-inducing factor pathway was specifically activated in these inflammatory cells. Furthermore, monocyte/macrophage subcluster analysis revealed an increase of chemokine C-C-motif receptor 2 (CCR2)^+^ proinflammatory macrophages but a decrease of M2-like macrophages. Notably, S100a9 expression was significantly upregulated in both granulocytes and CCR2^+^ proinflammatory macrophages, accompanied by increased chromatin accessibility at the promoter region specifically in CCR2^+^ macrophages. Given its restricted upregulation in these two key proinflammatory cell populations, we generated S100a9 global knockout mice to investigate its role in PAH. S100a9 deletion alleviated the pulmonary arterial remodeling and right ventricular dysfunction in PAH mice.

**Conclusion:**

In conclusion, this study established a comprehensive transcriptome and chromatin accessibility atlas for PAH mice and further indicated that activation of S100a9-expressing inflammatory cell might be associated with the development of PAH. Further researches are warranted to investigate the underlying mechanisms.

## Graphical Abstract

**Figure:**
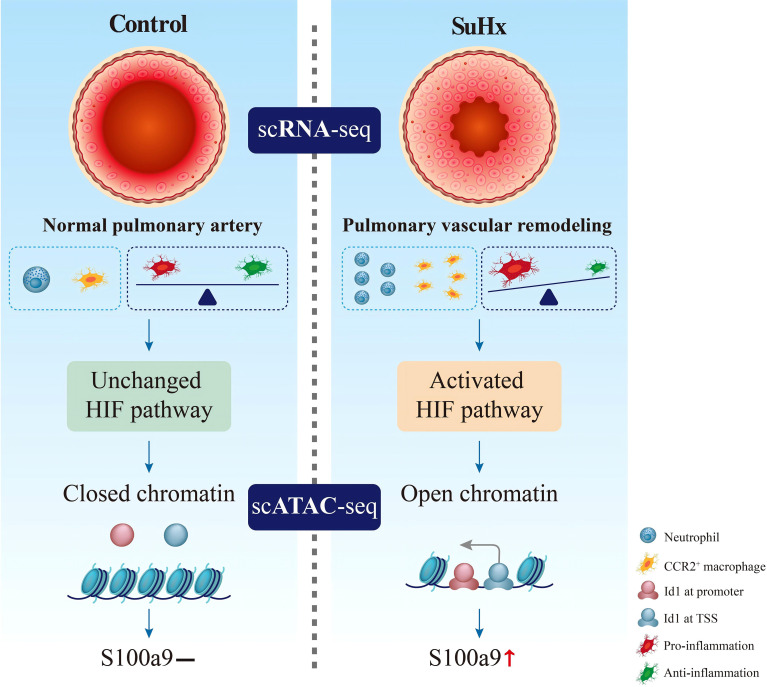
Diagram of the key mechanisms in PAH. In PAH mice, the number of proinflammatory cells in the lungs is increased, while the number of anti-inflammatory cells is decreased. The hypoxia-inducing factor pathway is specifically activated in granulocytes and monocytes/macrophages in PAH mice. In CCR2^+^ proinflammatory macrophages, the accessibility of S100a9 in the promoter region and transcriptional start site was increased, which could be regulated by Id1 in PAH mice. S100a9 may play a critical role in the development of pulmonary vascular remodeling and pulmonary hypertension.

## Introduction

Pulmonary hypertension (PH) is an intractable clinical syndrome with multiple etiologies, including heredity, left heart disease, lung disease and/or hypoxia, and chronic thromboembolism (1). Pulmonary arterial hypertension (PAH), categorized as group 1 of PH, is one of the leading causes of PH worldwide (2). During the development of PAH, pulmonary vascular remodeling is the main pathological feature, resulting from endothelial dysfunction, inflammatory cell activation, smooth muscle cell (SMC) proliferation and proinflammatory phenotypic transformation, fibroblast activation and extracellular matrix (ECM) deposition (3). Although great advances have been made in the pathophysiology and molecular mechanisms of PAH, the core mechanism of the development of PAH remains unknown. Therefore, a comprehensive cellular and molecular profile of the pathology of PAH is urgently needed to make a breakthrough in exploring the critical underlying mechanism and developing effective therapeutic targets.

By using single-cell RNA sequencing (scRNA-seq), recent studies have revealed the immune cell composition of idiopathic PAH in humans (4) and the inflammatory landscape of two rat models of PAH (5). Furthermore, a single-cell assay for transposase-accessible chromatin sequencing (scATAC-seq) has been used to measure chromatin accessibility. The role of chromatin accessibility in the promotion or inhibition of disease progression is of great importance for understanding the mechanisms involved and finding therapeutic targets (6). Moreover, a joint scRNA-seq and scATAC-seq analysis could establish a framework for understanding how chromatin accessibility regulates transcription (7). However, the single-cell chromatin accessibility landscape of PAH has not yet been constructed, and a comprehensive overview of the various cell types and their activation status in the PAH lung is lacking.

In this study, scRNA-seq and scATAC-seq were performed to establish a transcriptional and chromatin accessibility atlas in a PAH model, and the key molecules and regulatory mechanisms involved in pulmonary vascular remodeling were explored through the joint scRNA-seq and scATAC-seq analysis. These results may provide a new theoretical basis and intervention targets for the prevention and treatment of PAH.

## Methods

### Animals treatments

Adult male C57BL/6J mice (8-10 weeks) were used under institutional guidelines (Laboratory Animal Welfare and Ethics Committee of Third Military Medical University, ethics approval number: AMUWEC20210293). During the study, mice were initially anesthetized with 4% isoflurane and maintained under 2% isoflurane. Euthanasia was performed on all animals by intraperitoneal injection of an overdose of sodium pentobarbital (150 mg/kg). Mice received weekly intraperitoneal injections of SU5416 (20 mg/kg, Sigma–Aldrich) during hypoxia (10% O_2_, 5 weeks). Age-matched controls received vehicle under normoxia. *S100a9*^-/-^ mice (Shanghai Southern Model Biology) were treated under the same conditions as the experimental group. In our study, each experimental group consisted of a sample size of 6, with the exception of the sequencing experiments, where each group comprised 4 mice. All mice were housed at 23 ± 2 °C with 12-h light/dark cycles.

### Tissue processing & single-nucleus suspensions

Lungs from 8 mice/group were dissected in ice-cold DMEM/5% FBS. Tissues were minced and digested using a Mouse Lung Dissociation Kit (Miltenyi Biotec), followed by PBS perfusion to remove blood. Enzymatic dissociation was performed in gentleMACS™ C tubes (program: 37C_m_LDK_1). Suspensions were filtered (100-μm MACS SmartStrainer), centrifuged, and resuspended. Quality control ensured: viability >90%, clumps <15%, live cells 300-600/μl, diameter 5-40 μm, nucleation >70%.

### Single-cell RNA-sequencing

Single-Cell RNA-seq libraries were constructed with Chromium Next GEM 3' v3.1 kits (10× Genomics; ~9,000 nuclei/lane) (8). Sequencing (NovaSeq 6000, Illumina) generated 50,000 reads/cell. Data were aligned to mm10 (Cell Ranger v6.0.1) and filtered for: 1,000–6,000 genes/cell, 2,000–40,000 UMIs, mitochondrial genes <5%, and ≥3 expressed gene (9). Batch correction used Harmony (v1.0), yielding 52,044 cells for analysis (**Supplementary Tables 1**, **2**).

### Cell type identification

Seurat v4.0.0 performed UMAP dimensionality reduction and clustering. Clusters were manually annotated using canonical markers (**Supplementary Table 3**). The rgl package (v0.99.16) aided visualization. Differentially expressed genes (DEGs) between clusters were identified using Seurat’s FindMarkers (Wilcoxon test, LogFC threshold 0.05; |logFC| > 0.5 defined DEGs).

### DEG module analysis

The ‘AverageExpression’ function was utilized to calculate the average expression of up- and down- regulated DEGs across 9 cell types in Con and PAH groups. This analysis provided a comprehensive overview of gene expression patterns and facilitated the identification of key regulatory genes involved in PAH. DEGs were clustered using the pheatmap R package (version 1.0.12), employing both hierarchical clustering (‘hcluster’) and k-means clustering (‘kmeans_k’ function) to form discrete modules of distinct expression profiles (10). Six modules were identified through this method, which were subsequently analyzed for functional enrichment.

### Pathway enrichment and KEGG analysis

Pathway enrichment and KEGG analyses were conducted on the top 200 DEGs per cluster using the clusterProfiler package (11). Significance thresholds were set at p < 0.05 and q < 0.05 to ensure robust findings. The top five enriched terms for each cluster were visualized as heatmaps using ggplot2 and enrichplot, providing a clear depiction of the most prominent pathways.

### Cell–cell communication

The CellPhoneDB data library (https://www.cellphonedb.org/) was used for cell–cell interactions. Ligand-receptor pairs expressed in >10% of any cell type (Con or PAH) were considered. Pairs with p < 0.01 were used to predict significant interactions between cell types (10).

### Pseudotime trajectory analysis

Monocle2 constructed pseudotime trajectories to explore biological processes and key genes, defining start/end clusters as described (12). Highly variable genes were used to sort cells in pseudotime order. Trajectories and gene expression heatmaps (genesorteR plotMarkerHeat) visualized results. Pathway enrichment and GO analysis followed prior methods (13).

### Transcription factor network analysis

PySCENIC (v0.10.3) inferred TF regulatory networks using default parameters and the mm10 cisTarget database. Enriched motifs, target genes (regulons), and regulon activity were identified. Cytoscape (v3.4.0) visualized networks.

### Single-cell ATAC-sequencing

Nuclei from the same cell suspension employed in scRNA-seq were isolated and washed following 10× Genomics instructions, and scATAC-seq libraries were built using Chromium Single Cell ATAC v1.1 kits. Nuclei were counted (Countstar Rigel S2), resuspended in buffer, and libraries constructed using Chromium Chip E (Product Code 1000156) and ATAC Library kits (Product Code 1000110). Then, we generated the corresponding library (CapitalBio Technology, Beijing). NovaSeq sequencing followed. Reads aligned to mm10 using Cell Ranger ATAC (v2.0.0). Signac (v1.6.0) analyzed the filtered peak matrix. Cells were filtered based on: fragments in peaks (1,000-50,000), %reads in peaks (>15), blacklist ratio (<0.001), nucleosome signal (<4), and mitochondrial ratio (<0.25). The nucleosome signal score and transcriptional start site (TSS) enrichment scores were calculated.

### Differentially accessible sites and cell type specificity scores

A Seurat object containing the peak matrix and fragment file was created per sample. The enrichment of Tn5 insertion site numbers was quantified at TSS and cells with TSS score ≤1 or <500 peaks were removed. Frequency-inverse document frequency (TF-IDF) normalization, feature selection, and dimensionality reduction were applied. UMAP visualized graph-based clustering and nonlinear dimension reduction. ClusterProfiler performed GO analysis on annotated genes. ChromVAR determined TF activities per cell (14). Signac created the motif-peak matrix (CreateMotifMatrix).

### Cis-regulatory interaction prediction

Cicero predicted cis-regulatory interactions within the Monocle 3 framework using scATAC-seq data. It calculated a “Cicero accessibility” score (-1 to 1) for peak pairs within a user-defined distance, with higher scores indicating greater co-accessibility.

### Label transfer from scRNA-seq to scATAC-seq

The scRNA-seq and scATAC-seq datasets integration followed established methods (15): 1) Signac calculated the gene activity matrix for scATAC-seq cells; 2) Canonical correlation analysis identified transfer anchors between scATAC-seq (gene activity) and scRNA-seq datasets; 3) Cell type labels were transferred from scRNA-seq to scATAC-seq.

### Fulton index

To evaluate the changes in the right ventricle (RV), hearts were excised with lungs and placed in PBS for separation. After trimming auricles and excess vasculature, the RV and left ventricle plus septum (LV+S) were dissected, blotted dry, weighed, and the Fulton index calculated as RV/(LV+S).

### H & E staining

Mouse lung tissues were paraffin-embedded, sectioned at 6 μm, and stored at room temperature. Sections were stained with hematoxylin and eosin. ImageJ measured outer and inner vessel wall diameters; wall thickness (%) was calculated as [(outer diameter - inner diameter)/outer diameter] × 100.

### Immunofluorescence staining

Frozen lung sections were stained with mouse anti-α-SMA (1:200, Abcam) and rabbit anti-CD31 (1:200, Abcam); frozen heart sections with rabbit anti-WGA (1:500, Abcam). Briefly, slides were fixed (PFA, 30 min), washed (PBS 3×5 min), blocked (30 min), incubated with primary antibodies (4 °C, overnight), followed by species-matched Cy3/Alexa Fluor 488 secondary antibodies (1:500, Invitrogen), and counterstained with DAPI (Beyotime, Shanghai China). Images were acquired via confocal microscopy. Per mouse, 60-80 microvessels (20-70 μm diameter) adjacent to alveolar ducts/alveoli were classified as nonmuscularized (no muscle), partially muscularized (crescent muscle), or fully muscularized (complete medial cuff). The percentage of muscularized vessels (partial + full) per total vessels was calculated.

### Pulmonary angiogram

Anesthetized mice underwent thoracotomy and laparotomy. Heparin sodium was injected via the RV. A PE-20 catheter was inserted into the main pulmonary artery via the RV and secured. Lungs were perfused sequentially with PBS (~0.05 mL/min until blanched) and Microfil (MV-122, Flow Tech Inc.; 600 µL Microfil + 750 µL diluent + 67.5 µL curing agent, added immediately before use; ~0.05 mL/min). Perfusion was completed within 15 min. Lungs were stored at 4 °C overnight (wet paper), dissected, washed in PBS (RT, 15 min shaking), and gradient-dehydrated. Vascular branching (total length, number of branches, connections) within the imaged region was quantified using ImageJ.

### Echocardiographic measurements and hemodynamics

Following anesthesia, mice were positioned supine and the chest area was depilated. Echocardiography was performed first: the ultrasound probe was adjusted to obtain clear apical 4-chamber views. Images capturing more than 3 consecutive cardiac cycles were recorded and saved. The right ventricular fractional area change (RVFAC%) was calculated offline as: [(RV diastolic area - RV systolic area)/RV diastolic area] × 100.Immediately after echocardiography, invasive hemodynamic measurements were obtained. A 1F Millar pressure catheter (SPR-1000) connected to a data acquisition system (AD Instruments, PL3508) was carefully advanced into the right ventricle (RV) under real-time pressure monitoring to confirm placement and record the characteristic RV pressure waveform. For pulmonary artery (PA) pressure assessment, the catheter was then advanced further into the PA to obtain the PA pressure signature. Continuous pressure recordings spanning several respiratory cycles were acquired and saved for subsequent analysis.

### Statistics and reproducibility

Data are expressed as mean ± standard deviation (SD). The normality of the data distribution was assessed via Kolmogorov-Smirnov test. For data that met the criteria for parametric analysis, including homogeneity of variance (p>0.05), statistical comparisons were conducted using either the Student’s t-test for 2-group comparisons. In case where data did not meet parametric assumptions, the Kruskal-Wallis test was employed. A p-value of less than 0.05 was considered indicative of statistical significance. All statistical analyses were performed using GraphPad Prism 8 software.

## Results

### The lung transcriptional landscape in PAH mice

**Figure 1:**
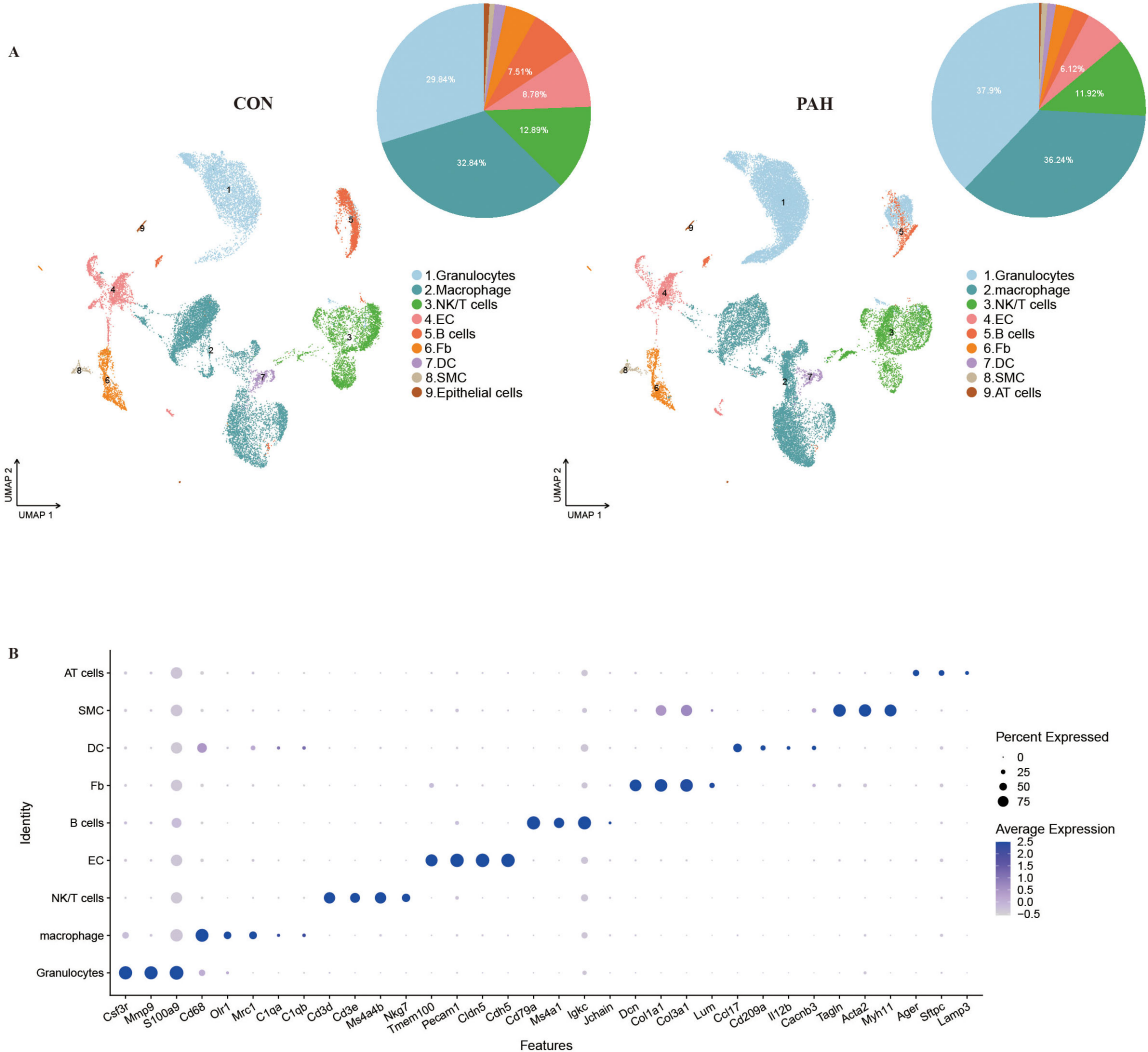
Integrated scRNA-seq dataset. **(A)** Visualization analysis of data dimensionality reduction by UMAP, where different numbers and colors represent different cell types, with the corresponding note on the right. **(B)** Heatmap of the top 3 marker genes corresponding to each cell type in mouse lung tissue, where each row represents one cell type, and each column represents the expression of one gene.

To construct a high-resolution molecular atlas of PAH and explore the key mechanisms involved, we constructed an PAH model in mice by using 10% oxygen and performing SU5416 injection for 5 weeks (16). Thereafter, cell suspensions from the lungs of control mice (Con mice, n=4) and experimental mice (PAH mice, n=4) were subjected to scRNA-seq and scATAC-seq, and a single-cell transcriptomic atlas and single-cell chromatin accessibility atlas were obtained. Then, cell type identification, cell–cell interaction analysis, transcription factor identification and pathway gene analysis were performed (**Supplementary Figure 1**). After quality control, a total of 52,044 cells were analyzed. The visualization analysis of data dimensionality reduction was performed by using uniform manifold approximation and projection (UMAP), and 9 cell clusters were identified according to their marker genes, including granulocytes (17,960 cells, mainly expressing S100a9, Ngp and S100a8), monocytes/macrophages (16,625 cells, mainly expressing Lyz2 and Mrc1), NK/T cells (7,383 cells, mainly expressing Gzma and Cd3g), endothelial cells (ECs, 3,479 cells, mainly expressing Cldn5 and Tmem100), B lymphocytes (3,093 cells, mainly expressing Cd79a and Cd79b), fibroblasts (2,208 cells, mainly expressing Dcn), dendritic cells (DCs, 693 cells, mainly expressing Ccl17), SMCs (408 cells, mainly expressing Tagln) and epithelial cells (195 cells, mainly expressing Ager and Sftpc) (**Figures 1A, B**; **Supplementary Table 3**). Through cell proportion analysis, the distribution of various cell types in the Con and PAH groups was revealed. Monocytes/macrophages constituted the most abundant population in our dataset, accounting for 32.84% of all cells, granulocytes accounted for 29.84%, NK/T cells for 12.89%, ECs for 8.78%, and B lymphocytes for 7.51%. After SuHx (SU5416+hypoxia) exposure, we found that the numbers of granulocytes and monocytes/macrophages were significantly increased in the PAH group, while the numbers of ECs and B lymphocytes were significantly decreased (**Figure 1A**).

### Single-cell chromatin accessibility atlas in the lungs of PAH mice

**Figure 2:**
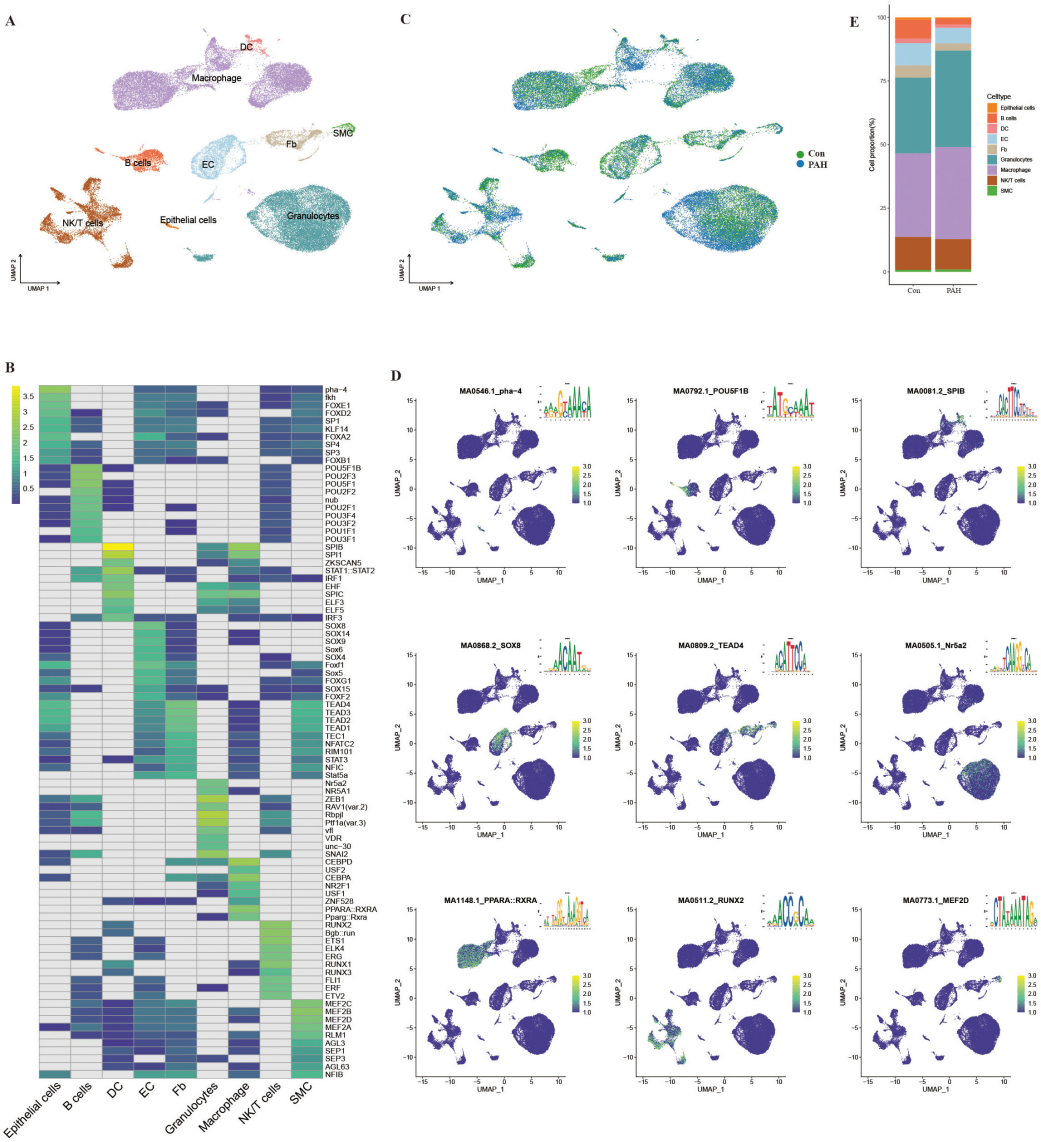
Integrated scATAC-seq dataset. **(A)** Visualization analysis of data dimensionality reduction by UMAP, where different numbers and colors represent different cell types. **(B)** Transcription factor heatmap of the top 10 transcription factors corresponding to each cell type. **(C)** Single-cell chromatin accessibility atlas of lung tissue from the Con group (green) and PAH group (blue) by UMAP. **(D)** UMAPs of motif activity for the top transcription factor corresponding to each cell type. **(E)** Relevant cell proportion analysis of the Con group and PAH group.

In this study, single nuclei of lung cells were prepared and treated with transposase, followed by barcoding and sequencing. After filtration, 46,762 nuclei were obtained from the Con group (22,907) and PAH group (23,855). scATAC-seq was used to divide the total nuclear population into 9 cell clusters by UMAP analysis, and cell identity was determined by transferring the labels among the nearest scRNA-seq neighbors to scATAC-seq cells (**Supplementary Figure 2**; **Figure 2A**). As shown in the transcription factor heatmap, epithelial cells specifically expressed transcription factors including pha-4, fkh, FOXE1, FOXD2 and SP1; B lymphocytes showed enrichment of transcription factors including POU5F1B, POU2F3, POU5F1, POU2F2 and Nub; DCs showed enrichment of transcription factors including SPIB, SPI1, ZKSCAN5, STAT1::STAT2 and IRF1; ECs showed enrichment of transcription factors including SOX8, SOX14, SOX9, SOX6, and SOX14; and fibroblasts showed enrichment of transcription factors including TEAD4, TEAD3, TEAD2, TEAD1, and TEC1. Granulocytes showed enrichment of transcription factors including Nr5a2, NR5A1, ZEB1, RAV1(var.2), and Rbpjl, while monocytes/macrophages showed enrichment of transcription factors including CEBPD, USF2, CEBPA, NR2F1, and USF1. NK/T cells showed enrichment of transcription factors including RUNX2, Bgb::run, ETS1, ELK4, and ERG, and SMCs showed enrichment of transcription factors including MEF2C, MEF2B, MEF2D, MEF2A, and RLM1 (**Figures 2B, D**). The distribution of single-cell chromatin accessibility patterns in the Con and PAH groups is shown in **Figure 2C**. We found that the number of granulocytes and monocytes/macrophages in the PAH group was greater than that in the Con group (**Figure 2E**).

### Molecular characterization of PAH pathology

**Figure 3:**
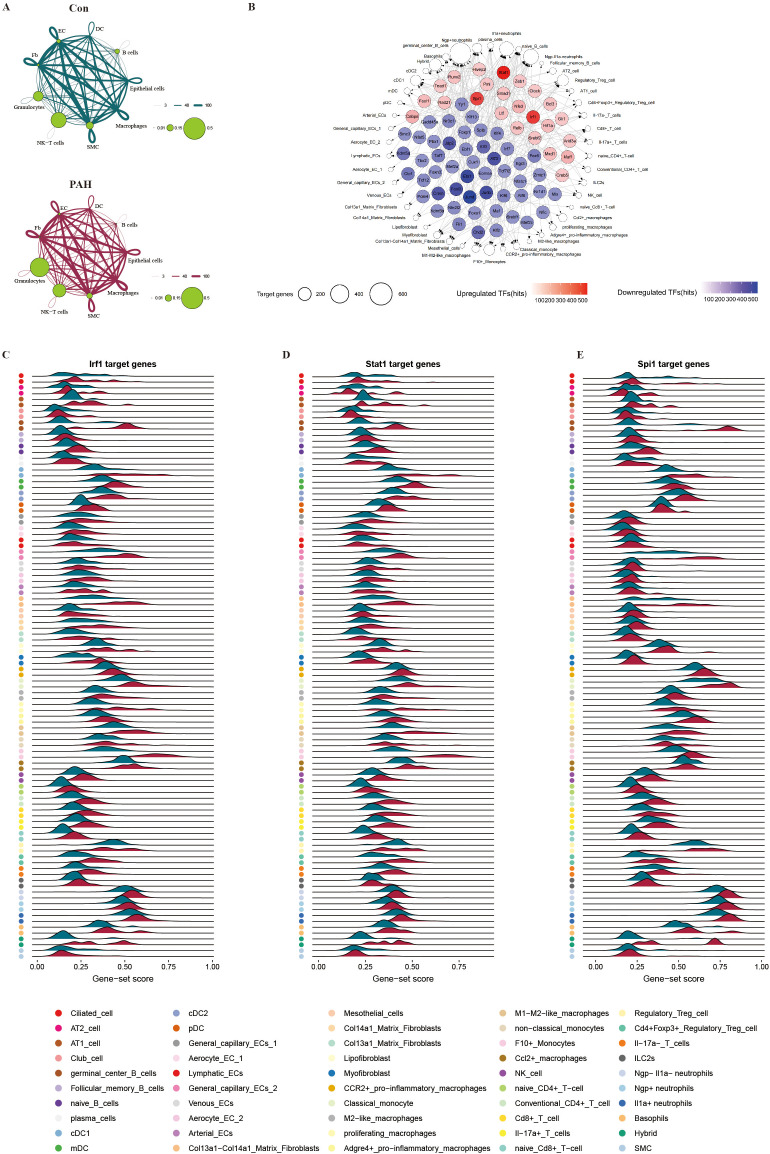
Joint scRNA-seq and scATAC-seq analysis to identify the characteristics of PAH pathology. **(A)** Cell–cell interactions in lung tissues from the Con group (upper) and PAH group (bottom). **(B)** SCENIC analyses identified the differential core transcription factors between Con group and PAH group. The outer nodes represent different cell types, and their size represents the number of target genes involved in that cell type, while the inner nodes represent the up- and downregulated transcription factors. The intensity of the color indicates the number of target genes that are regulated by these transcription factors. Ridge maps showing the gene-set scores of targets genes of Irf1 **(C)**, Stat1 **(D)** and Spi1 **(E)** in different cell types.

**Figure 4:**
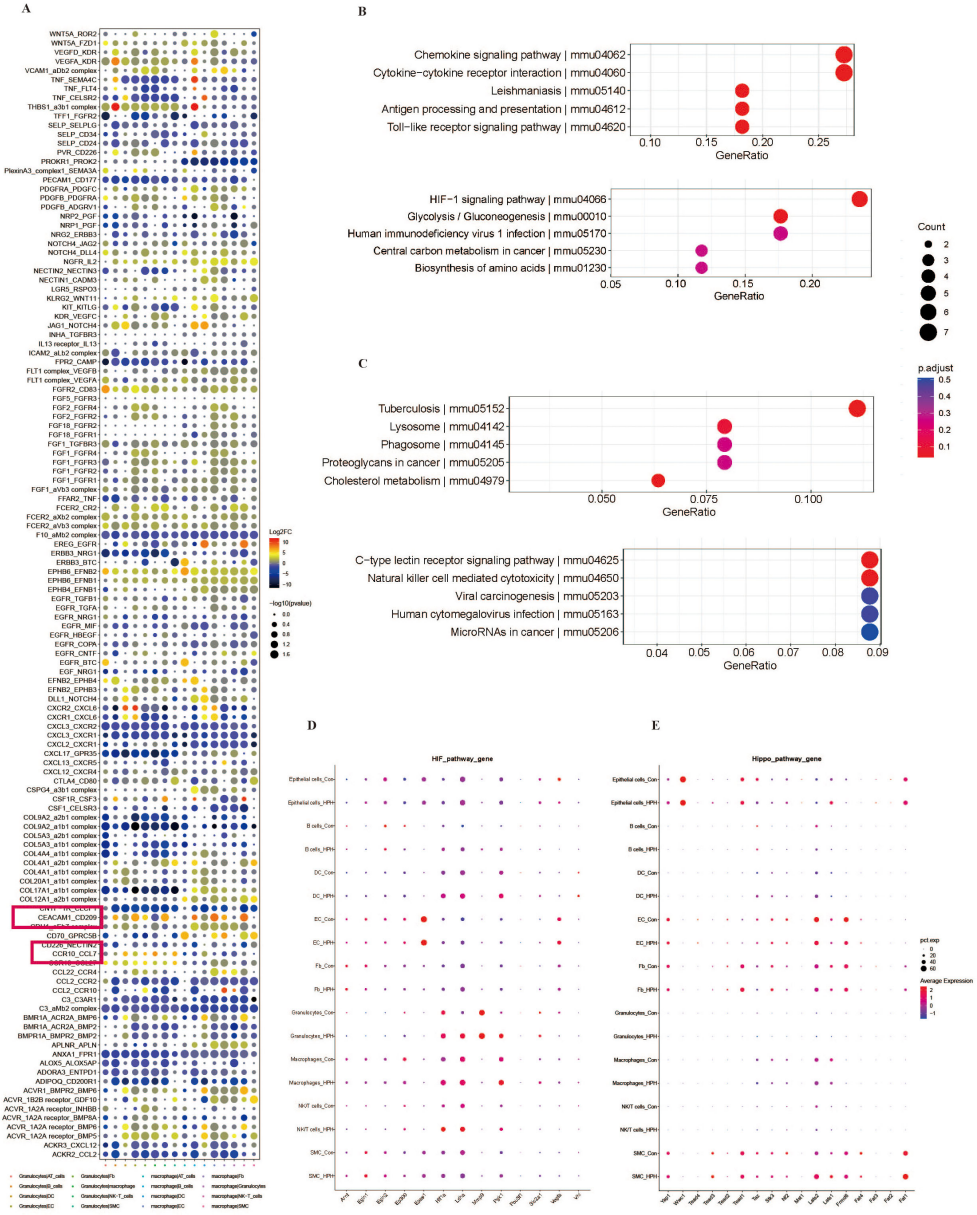
The molecular characteristics of PAH pathology. **(A)** Differential expression of ligand–receptor interactions, with each column as a set of cell types represented in different colors. **(B)** KEGG enrichment analysis of differential gene expression in 2 upregulated clusters. **(C)** KEGG enrichment analysis of differential gene expression in 2 downregulated clusters. **(D)** HIF and **(E)** Hippo pathway genes in nine subgroups of cells in the lung tissues of the Con group and PAH group.

The analysis of cell–cell interactions revealed that the frequency of granulocytes and the absolute number of granulocyte and monocyte/macrophage interactions were increased in PAH mice (**Figure 3A**). Furthermore, we used the UMAP dimensionality reduction visualization method to recluster these subtype cells and annotated these clusters according to their marker genes. We ultimately obtained 4 subsets of epithelial cells, including Type I alveolar epithelial cells (AT1 cells), AT2 cells, Ciliated cells and Club cells (**Supplementary Figure 3A**); 4 subsets of B lymphocytes, including Follicular memory B cells, Germinal center B cells, naive B cells and plasma B cells (**Supplementary Figure 3B**); 4 subsets of DCs including classical DC1, classical DC2, migratory DC and plasmacytoid DC (**Supplementary Figure 3C**); 7 subsets of ECs, including Aerocyte EC1, Aerocyte EC2, Arterial ECs, General capillary EC1, General capillary EC2, Lymphatic ECs and Venous ECs (**Supplementary Figure 3D**); 6 subsets of fibroblasts, including Col13a1 matrix fibroblasts, Col14a1 matrix fibroblasts, Col13a1-Col14a1 matrix fibroblasts, lipofibroblasts, mesothelial cells and myofibroblasts (**Supplementary Figure 3E**); 10 subsets of natural killer (NK)/T cells, including Cd4^+^Foxp3^+^ regulatory Treg cells, Cd8^+^ T cells, conventional Cd4^+^ T cells, IL17a^-^ T cells, IL17a^+^ T cells, Type 2 innate lymphoid cells (ILC2s), naive Cd4^+^ T cells, naive Cd8^+^ T cells, NK cells and regulatory Treg cells (**Supplementary Figure 3F**); 5 subsets of granulocytes, including basophils, hybrid granulocytes, IL1α^+^ neutrophils, Ngp^-^IL1α^-^ neutrophils, and Ngp^+^ neutrophils (**Supplementary Figure 3G**); and 9 subsets of monocytes/macrophages, including CCR2^+^ pro-inflammatory macrophages, M2-like macrophages, Adgre4^+^ pro-inflammatory macrophages, F10^+^ monocytes, classical monocytes, M1/M2-like macrophages, proliferative macrophages, nonclassical monocytes, and Ccl2^+^ macrophages (**Supplementary Figure 3H**). Thereafter, SCENIC analyses were performed to identify candidate transcription factors that control differentially expressed genes in different cell types. The expression levels of Stat1, Spi1 and Irf1 were significantly upregulated, and Jund and Ets1 were the top transcriptional regulators of downregulated genes after SuHx exposure (**Figure 3B**). Moreover, the expression levels of Irf1, Stat1 and Spi1 were significantly increased in monocytes/macrophages and granulocytes (**Figures 3C–E**). The enriched ligand–receptor interactions across granulocytes and monocytes/macrophages included CEACAM1–CD209 and CCR10–CCL7 (**Figure 4A**), which were mainly involved in inflammatory responses. A modularized analysis of differentially expressed genes (DEGs) was performed to investigate cell type-specific transcriptional characteristics during PAH pathogenesis. We identified a total of 18,106 DEGs in 9 cell types and 50 cell subclusters and found significant differences in monocytes/macrophages and granulocytes. We identified 6 DEG clusters with upregulated expression (**Supplementary Figure 4A**) and 6 DEG clusters with downregulated expression (**Supplementary Figure 4B**). Among these clusters, upregulated cluster 1 (mainly chemokine signaling pathway and cytokine–cytokine receptor interaction) and upregulated cluster 5 (mainly HIF-1 signaling pathway) were mainly enriched in granulocytes and monocytes/macrophages (**Figure 4B**); we also found that downregulated clusters 3 and 4, enriched in granulocytes and monocytes/macrophages, were associated with lysosome and phagosome effects, the c-type lectin receptor signaling pathway and cytotoxic effects (**Figure 4C**). The other upregulated clusters, including cluster 3 (mainly TNF signaling pathway and NOD-like receptor signaling pathway), cluster 6 (mainly cytokine–cytokine receptor interaction), cluster 4 (mainly cytosolic DNA-sensing pathway) and cluster 2 (mainly osteoclast differentiation and carbon metabolism), are shown in **Supplementary Figures 5A–D**. Other downregulated clusters are shown in **Supplementary Figures 5E–H**. We further analyzed the signaling pathways in different cell types involved in PAH, and the results showed that hypoxia triggered the expression of hypoxia-inducing factor (HIF) pathway genes (Hif1α, Ldha and Pgk1), especially in granulocytes and monocytes/macrophages (**Figure 4D**), suggesting that the HIF signaling pathway was mainly activated in granulocytes and macrophages during PAH development. However, the Hippo signaling pathway, the EMT signaling pathway, the TGF signaling pathway, the WNT signaling pathway and the NOTCH signaling pathway were not activated in granulocytes and macrophages during PAH development. (**Figure 4E**; **Supplementary Figure 6A–D**).

### Aberrant activation of granulocytes in PAH mice

**Figure 5:**
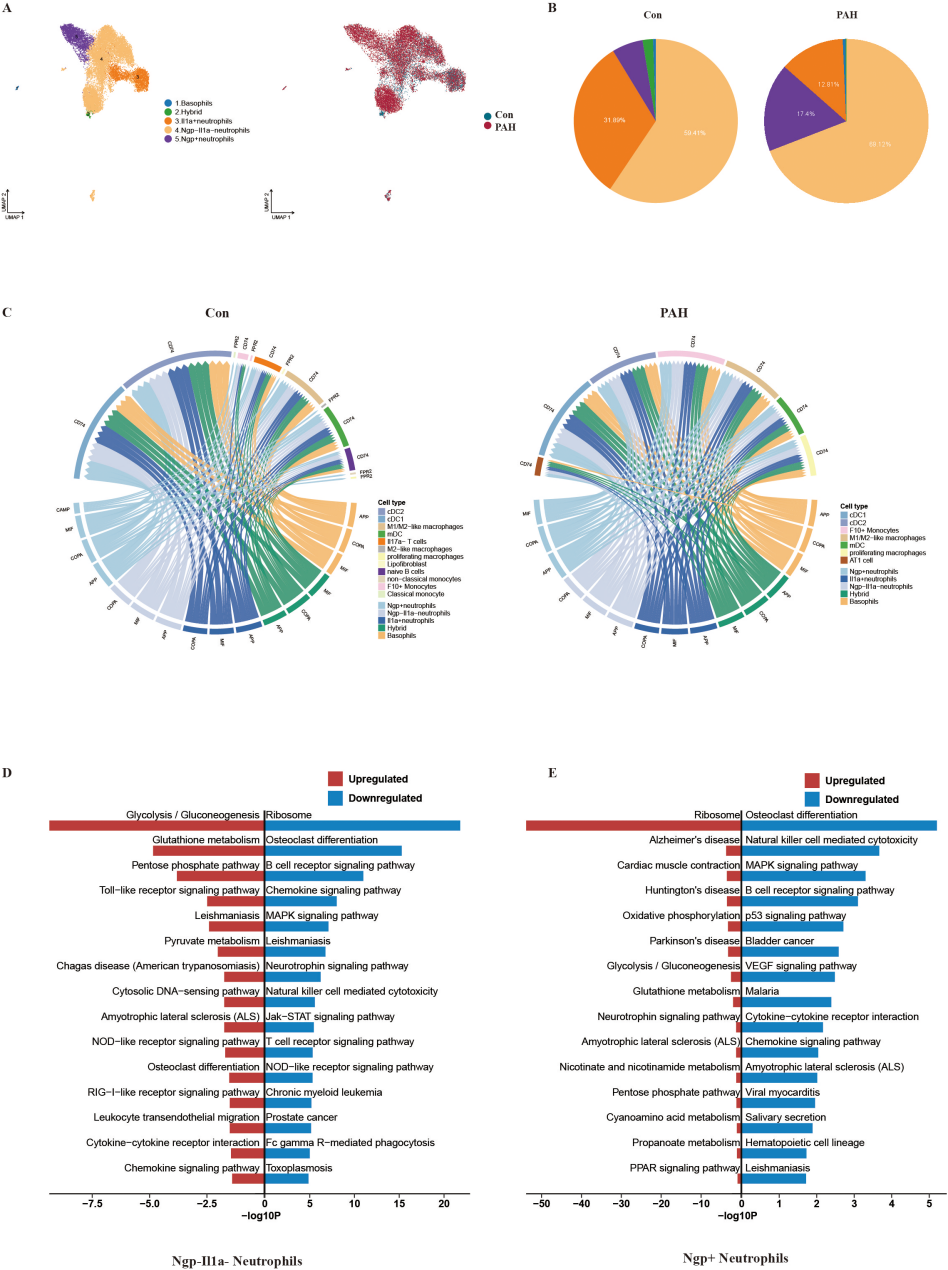
Characteristics of granulocytes between the Con and PAH groups by scRNA-seq. **(A)** Visualization analysis of data dimensionality reduction by UMAP, different numbers and colors represent different cell types, and single-cell transcriptomic atlas of lung tissue from the Con group (blue) and PAH group (red) by UMAP. **(B)** Relevant cell proportion analysis of the Con group (left) and PAH group (right). **(C)** Circos analyses to establish granulocyte-centered communication, where granulocytic cells are the senders in the Con group (top) and PAH group (bottom), and edge thickness is proportional to edge weight. The edge color marks the source cell type. **(D)** KEGG enrichment analysis of differential pathways in Ngp^-^IL1α^-^ neutrophils. **(E)** KEGG enrichment analysis of differential pathways in Ngp^+^ neutrophils.

We further used the UMAP dimensionality reduction visualization method to recluster the granulocytes and obtained 5 subsets of granulocytes (**Figure 5A**). Consistent with previous studies (17–20), our data showed that Wfdc17, Ifitm1, Cstdc4 and Saft2 were mainly expressed in Ngp^-^IL1α^-^ neutrophils; Camp, Ltf and Ngp were mainly expressed in Ngp^+^ neutrophils; Ccrl2, Gadd45b, and Rsad2 were mainly expressed in IL1α^+^ neutrophils; Ccl3 and Gzmb were mainly expressed in basophils; and Cd74 and Igkc were mainly expressed in hybrid granulocytes (**Supplementary Figure 7A**). Using the regulon specificity score, we also revealed the top 5 transcription factors in each granulocyte subtype (**Supplementary Figure 8**). Compared with the Con group, the PAH group showed an increase in Ngp^-^IL1α^-^ neutrophils and Ngp^+^ neutrophils (**Figures 5A, B**). To investigate the association between granulocyte subclusters and other cell subclusters, we performed a connectomic analysis that mapped the average gene expression of each cell type to known ligand–receptor interactions in the NicheNet database (21). We first demonstrated that granulocytes presented a stronger association with monocytes/macrophages in PAH mice when they acted as sender cells (**Figure 5C**). However, when granulocytes were used as receivers, the association between granulocytes and goblet epithelial cells was obviously increased (**Supplementary Figures 9A, B**). Pseudotime trajectory analysis was used to construct the pseudotemporal locus of granulocytes and their temporal distribution, which showed that the branch terminals corresponded to IL1α^+^ neutrophils, Ngp^+^ neutrophils, basophils and hybrid granulocytes, while Ngp^-^IL1α^-^ neutrophils were found in all branches (**Supplementary Figures 7B, C**). We further tracked the dynamic differentiation state of each subcluster during PAH development. Ngp^-^IL1α^-^ neutrophils were mainly located in the upper left branch in the Con group but moved to the upper right branch and lower branch in the PAH group. However, Ngp^+^ neutrophils showed a significant increase in the upper right branch after SuHx exposure (**Supplementary Figure 7D**). Then, we focused on the analysis of DEGs and KEGG pathways in Ngp^+^ neutrophils and Ngp^-^IL1α^-^ neutrophils. We found that the main upregulated genes in Ngp^+^ neutrophils were Cstdc5, Stfa1, Stfa2 and Asprv1, while the main downregulated genes were Ppp1r3d, Fam32a, Hlx and Thbs1 after SuHx exposure (**Supplementary Figure 7E**). The main upregulated KEGG pathway was the ribosome pathway, and the main downregulated KEGG pathway was osteoclast differentiation (**Figure 5D**). In Ngp^-^IL1α^-^ neutrophils, Cstdc5, Stfa2, Stfa1 and Ifitm6 were mainly upregulated, and the main downregulated genes were Rbbp8, Lamp1, Ptges3, Fosb and Vps37b (**Supplementary Figure 7F**). The main upregulated KEGG pathway was glycolysis/gluconeogenesis, and the main downregulated KEGG pathway was the ribosome pathway (**Figure 5E**).

### Altered chromatin accessibility in granulocytes of PAH mice

**Figure 6:**
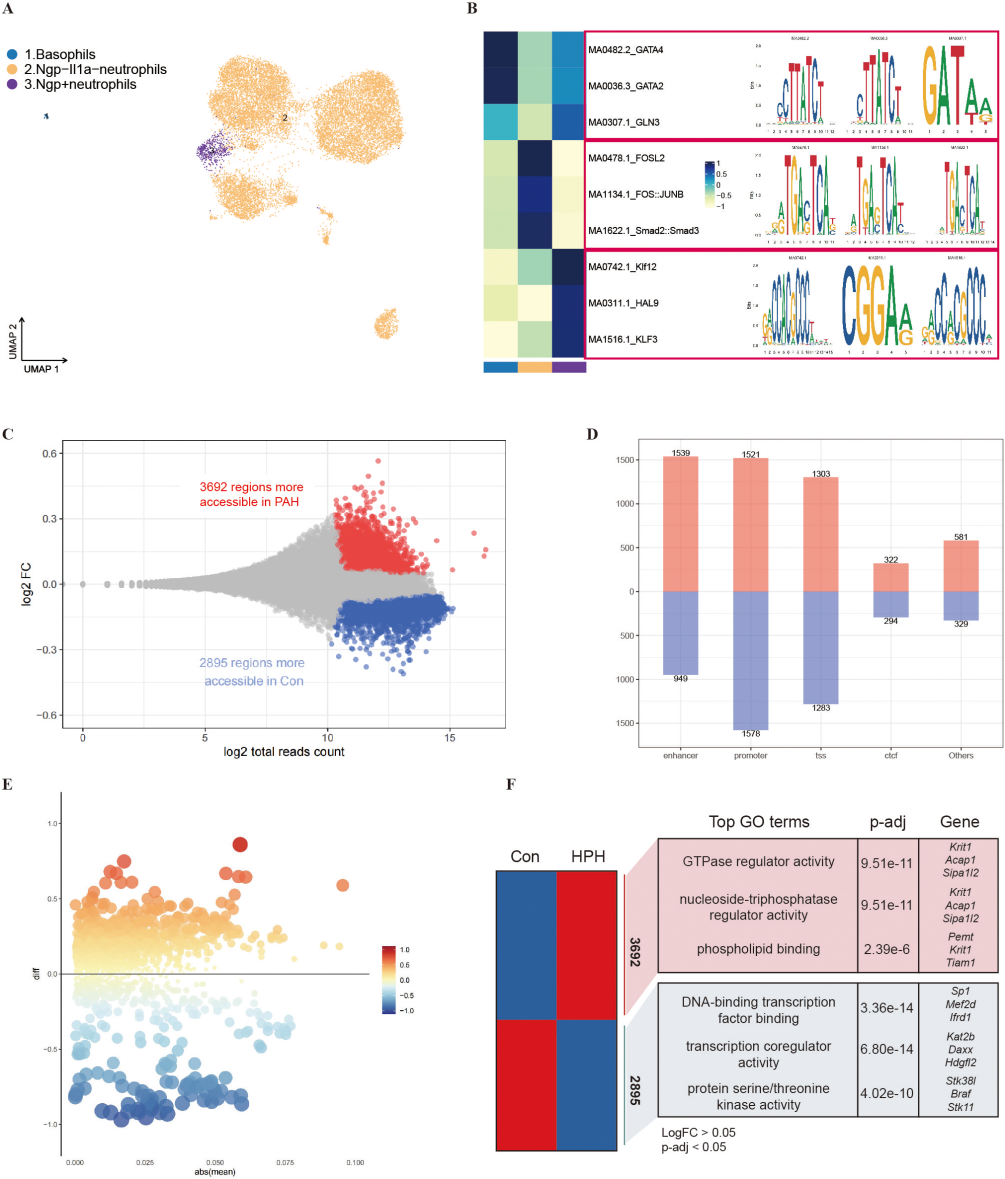
Alterations of chromatin accessibility in granulocytes revealed by scATAC-seq. **(A)** Single-cell chromatin accessibility atlas of granulocytes in mouse lung tissue obtained by UMAP, where each color represents one cell type. **(B)** Transcription factor heatmap of the top 3 transcription factors corresponding to each cell type and their annotated motif (right). **(C)** MA plot showing the differential open regions of granulocytes in the PAH (red) and Con (blue) group, and the specified number of areas was calculated. **(D)** The histograms show the number of open or closed regions at the promoters and enhancers of granulocytes in mouse lung tissue. **(E)** Transcription factors in differential open regions in enhancers, where transcription factors are plotted based on their gene coverage p value and whether they exceed (red) or under (blue). **(F)** GO pathways and their top genes in lung tissue from the Con and PAH groups.

Granulocytes were then reclustered by scATAC-seq and subsequent UMAP analysis, and cell identities were determined by transferring the labels among the nearest scRNA-seq neighbors to scATAC-seq cells. The granulocytes were divided into three categories: Ngp^-^IL1α^-^ neutrophils, Ngp^+^ neutrophils, and basophils (**Figure 6A** and **S10**). Ngp^-^IL1α^-^ neutrophils showed enrichment of transcription factors including FOSL2, FOS::JUNB, Smad2::Smad3, FOS::JUN, and FOSL1::JUN; Ngp^+^ neutrophils showed enrichment of transcription factors including Klf12, HAL9, KLF3, SP1, and ASG1; and basophils showed enrichment of transcription factors including GATA4, GATA2, GLN3, GATA3, and GATA5 (**Figure 6B**). Furthermore, the epigenomic landscapes of these granulocytes were analyzed by ATAC-seq. We found 3692 differentially accessible regions in PAH mice and 2895 differentially accessible regions in Con mice, suggesting that more accessible regions were opened after SuHx exposure (**Figure 6C**). Moreover, we found 1539 open regions and 949 closed regions among enhancers and 1521 open regions and 1578 closed regions among promoters (**Figure 6D**). The binding motifs in the ATAC peaks in promoter regions showed significantly different enrichment of motifs between the PAH group and the Con group. For example, NRF1, CAT8, and ASG1 were significantly upregulated in the PAH group (**Figure 6E**). The GO pathway analysis of these accessible regions showed that the regions with increased accessibility in the PAH group were related to the regulatory activity of GTase (Krit1, Acap1, Sipa1/2), the regulatory activity of triphosphate nucleotide enzymes (Krit1, Acap1, Sipa1/2) and phospholipid binding (Pemt, Krit1, Tiam1), whereas the regions with decreased accessibility were associated with DNA-binding transcription factors (Sp1, Mef2d, Ifrd1), transcription coregulatory factor activities (Kat2b, Daxx, Hdgfl2), and serine protein/threonine kinase activities (Stk38l, Braf, Stk11) (**Figure 6F**). We found that the accessible regions of the Con mice were related to apoptosis, the cytokine signaling pathway, the TNF signaling pathway and the MAPK signaling pathway (**Supplementary Figure 11A**), while the accessible regions of the PAH mice were related to leukocyte transendothelial migration, cell senescence, the Rap1 signaling pathway and the VEGF signaling pathway (**Supplementary Figure 11B**).

### Characterization of monocytes/macrophages in PAH mice

**Figure 7:**
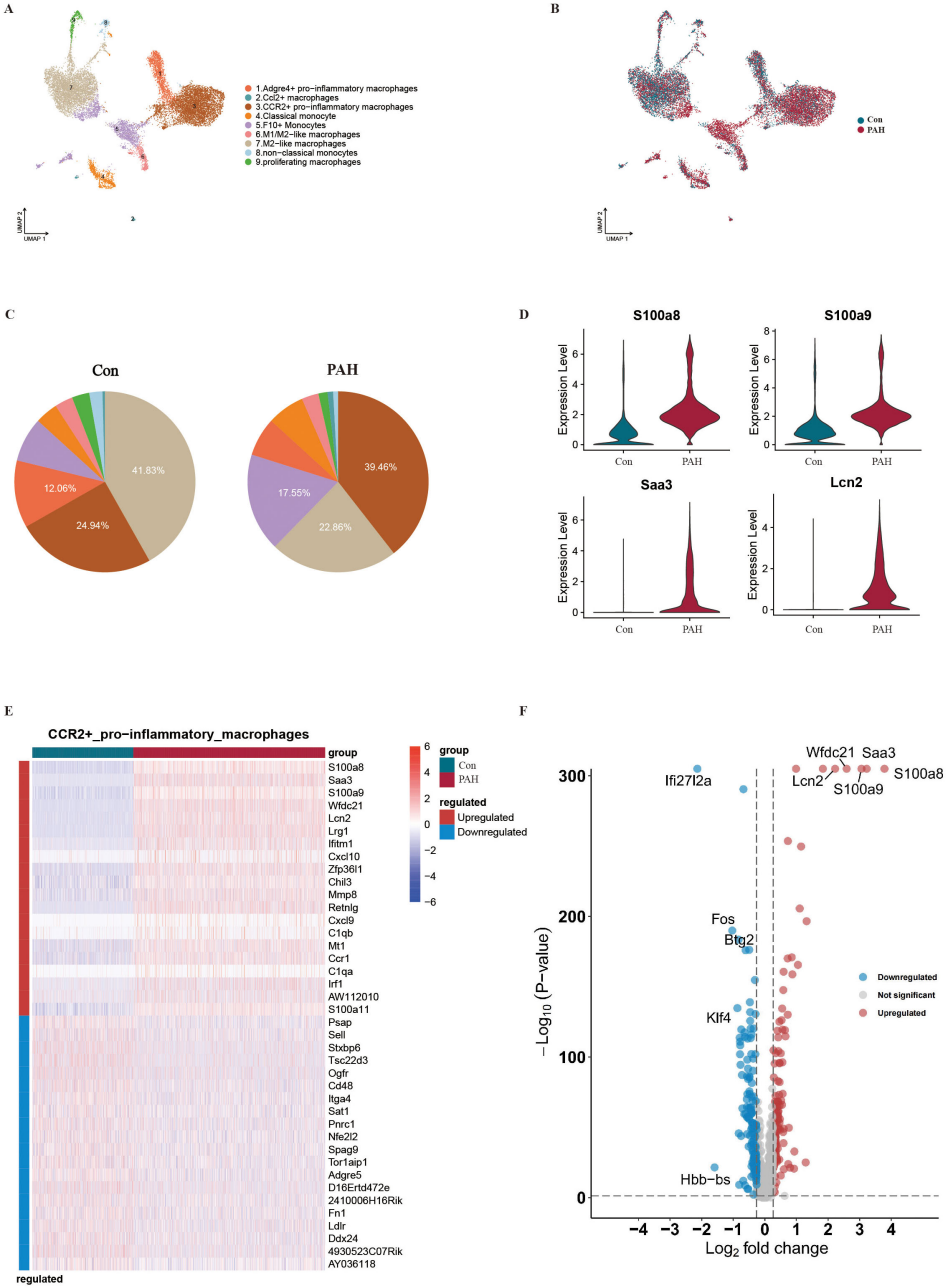
Characteristics of monocytes/macrophages between the Con and PAH groups revealed by scRNA-seq. **(A)** Visualization analysis of data dimensionality reduction by UMAP, where different numbers and colors represent different cell types. **(B)** Single-cell transcriptomic atlas of lung tissue from the Con group (blue) and PAH group (red) obtained by UMAP. **(C)** Relevant cell proportion analysis of the Con group (left) and PAH group (right). **(D)** Comparison of differential expression levels of the S100a8, S100a9, Saa3 and Lcn2 genes in lung tissue monocytes/macrophages between the Con group and PAH group. **(E)** Heatmap showing the genes that were differentially expressed in CCR2^+^ pro-inflammatory macrophages. **(F)** Volcano plots showing the genes that were differentially expressed in CCR2^+^ pro-inflammatory macrophages.

Monocytes/macrophages are one of the most abundant cell types in lung tissue. We conducted reclustering analysis of the monocyte/macrophage population and identified 9 subpopulations, including CCR2^+^ proinflammatory macrophages, M2-like macrophages, Adgre4^+^ proinflammatory macrophages, F10^+^ monocytes, classical monocytes, M1/M2-like macrophages, proliferative macrophages, nonclassical monocytes, and Ccl2^+^ macrophages (**Figure 7A**). Consistent with previous results (22–24), our data showed that Ace, Spn, Pou2f2, Adgre4 and Dusp16 were mainly expressed in proinflammatory Adgre4^+^ macrophages. Prg4, Alox15, Serpinb2, Saa3 and Slpi were mainly expressed in Ccl2^+^ macrophages, while Thbs1, Ly6c2, S100a4, Plac8 and Ccr2 were mainly expressed in CCR2^+^ proinflammatory macrophages. S100a9, Retnlg, S100a8, Ifitm1 and Lcn2 were mainly expressed in classical monocytes; Ccl5, Cxcl9 and AW112010 were mainly expressed in F10^+^ monocytes; and Ccl8, C1qa, Apoe and C1qb were mainly expressed in M1/M2-like macrophages. Plet1, Lpl, Cd9 and Ear2 were mainly expressed in M2-like macrophages; Epas1, Cldn5 and Ramp2 were mainly expressed in nonclassical monocytes; and Top2a, Mki67 and Ptprb were mainly expressed in proliferative macrophages (**Supplementary Figure 12A**). We also revealed the top 5 transcription factors in each macrophage subtype based on regulon specificity scores (**Supplementary Figure 13**). The comparison of cell distributions between the Con and PAH groups showed that the numbers of CCR2^+^ proinflammatory macrophages and F10^+^ monocytes were significantly increased after SuHx exposure, while the number of M2-like macrophages was decreased, suggesting that the balance of proinflammatory and anti-inflammatory mechanisms was disrupted by SuHx exposure (**Figures 7B, C**). Moreover, the expression levels of Saa3, S100a8, S100a9 and Lcn2 in the PAH group were higher than those in the Con group (**Figure 7D**). We further performed a pseudotime trajectory analysis, which showed that the main branch terminals corresponded to CCR2^+^ proinflammatory macrophages, M2-like macrophages, F10^+^ monocytes (**Supplementary Figure 12B** and **S14A**), and the pseudotemporal distribution of monocytes/macrophages in the Con and PAH groups (**Supplementary Figure 12C**). By using pseudotime trajectory-based differential expression analysis, we analyzed Arid5a, Dst, Mcm3, Neurl3 and Ptpn18 in different monocyte/macrophage subtypes (**Supplementary Figure 14B**). Furthermore, we found that CCR2^+^ proinflammatory macrophages showed upregulated expression of S100a8, Saa3, S100a9, Wfdc21 and Lcn2 and downregulated expression of Fos, Ifi27l2a, Btg2, Klf4 and Hbb-bs (**Figures 7E, F**). We also investigated the upregulated and downregulated DEGs in other monocyte/macrophage subtypes (**Supplementary Figure 15**).

### Altered chromatin accessibility in monocytes/macrophages of PAH mice

**Figure 8:**
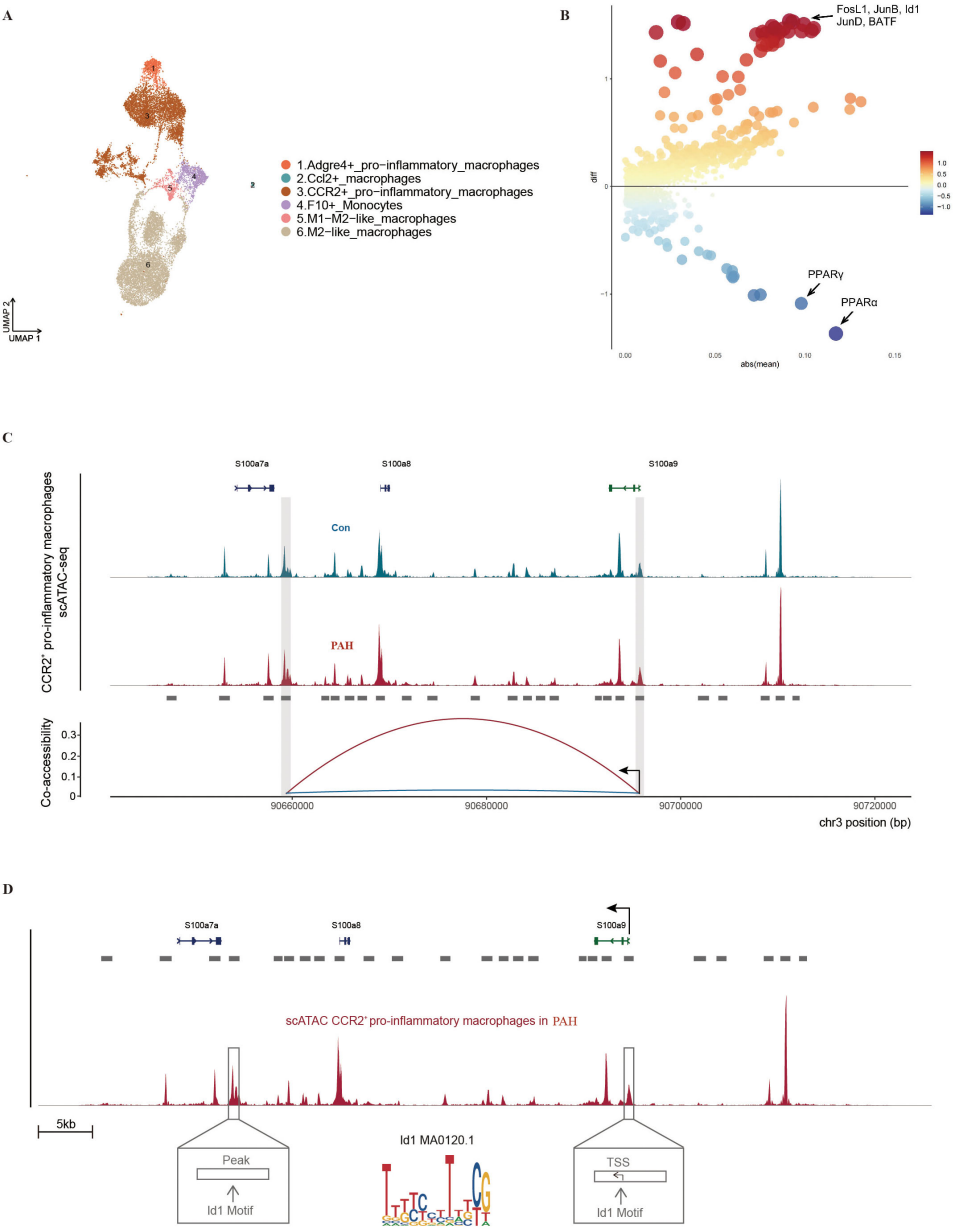
Alterations of chromatin accessibility in monocytes/macrophages revealed by scATAC-seq. **(A)** Single-cell chromatin accessibility atlas of monocytes/macrophages in mouse lung tissue obtained by UMAP, where each color represents one cell type. **(B)** Transcription factors in differential open regions in enhancers, where transcription factors are plotted based on their gene coverage p value and whether they exceed (red) or under (blue). **(C)** Coaccessibility analysis of S100a9 in CCR2^+^ pro-inflammatory macrophage from the Con (blue) and PAH (red) group. **(D)** Schematic representation of the S100a9 site with an average scATAC-seq signal in CCR2^+^ pro-inflammatory macrophages in PAH, and Id1 motif (MA0120.1) in the selected peak region and in the S100a9 promoter (TSS).

Based on scATAC-seq, monocytes/macrophages were then divided into six subclusters, including CCR2^+^ proinflammatory macrophages, M2-like macrophages, Adgre4^+^ proinflammatory macrophages, F10^+^ monocytes, M1/M2-like macrophages, and Ccl2^+^ macrophages (**Figure 8A**; **Supplementary Figure 16**). We found that CCR2^+^ proinflammatory macrophages showed enrichment of transcription factors including CTCF and YY1, and Adgre4^+^ proinflammatory macrophages showed enrichment of transcription factors including nhr-6, NR4A1 and NR2F2. M2-like macrophages showed enrichment of transcription factors including PPARA::RXRA, Pparg::Rxra and NR2F1 (var. 2), while M1/M2-like macrophages showed enrichment of transcription factors including NRL, Mafb and MEF2C. Ccl2^+^ macrophages showed enrichment of transcription factors including GATA3, GATA2, and GATA4, and F10^+^ monocytes showed enrichment of transcription factors including IRF8, IRF9, and IRF4 (**Supplementary Figure 17A**). Then, we found that 2569 accessible regions were opened in the PAH group, whereas 1809 accessible regions were opened in the Con group (**Supplementary Figure 18A**), 1392 open regions and 741 closed regions in enhancers, and 702 open regions and 711 closed regions in promoters (**Supplementary Figure 18B**). The GO pathway analysis of these accessible regions showed that the accessible regions of the PAH group were related to enzyme activator activity (Gm2a, Rin2, Sipa1/2), cytokine receptor activity (Il7r, Cd44, Il6st) and ubiquitin protein ligase (Axin2, Tgfbr1, Cd40). The accessible regions of the Con group were associated with transcription coregulatory factor activity (Pparg, Jup, Ppard), intermembrane lipid transport activity (Plekha8, Gltp, Abca1), and small GTases (Grasp, Kpnb1, Rac1) (**Supplementary Figure 17B**). Then, we analyzed the binding motifs on the ATAC peaks of these promoter regions and found that fo-like antigen 1 (FosL1), JunB, inhibitor of differentiation 1 (Id1), JunD and BATF binding sites were significantly upregulated in the PAH group, whereas the expression of PPARγ and PPARα was significantly decreased (**Figure 8B**). ScATAC-seq coaccessibility analysis showed that S100a9 in CCR2^+^ proinflammatory macrophages was significantly more coaccessible than that in the other subclusters after SuHx exposure (**Supplementary Figure 19**). Furthermore, the coaccessibility of the S100a9 promoter and transcription initiation site in CCR2^+^ proinflammatory macrophages was strongly increased after SuHx exposure (**Figure 8C**). Thereafter, we revealed that the Id1 motif (MA0120.1) bound to the S100a9 promoter and transcription initiation site (**Figure 8D**), and the IRF2 motif (MA0051.1) and STAT1::STAT2 motif (MA0517.1) bound to the S100a9 promoter (**Supplementary Figures 20A, B**).

### The effects of S100a9 deficiency on pulmonary vascular remodeling

**Figure 9:**
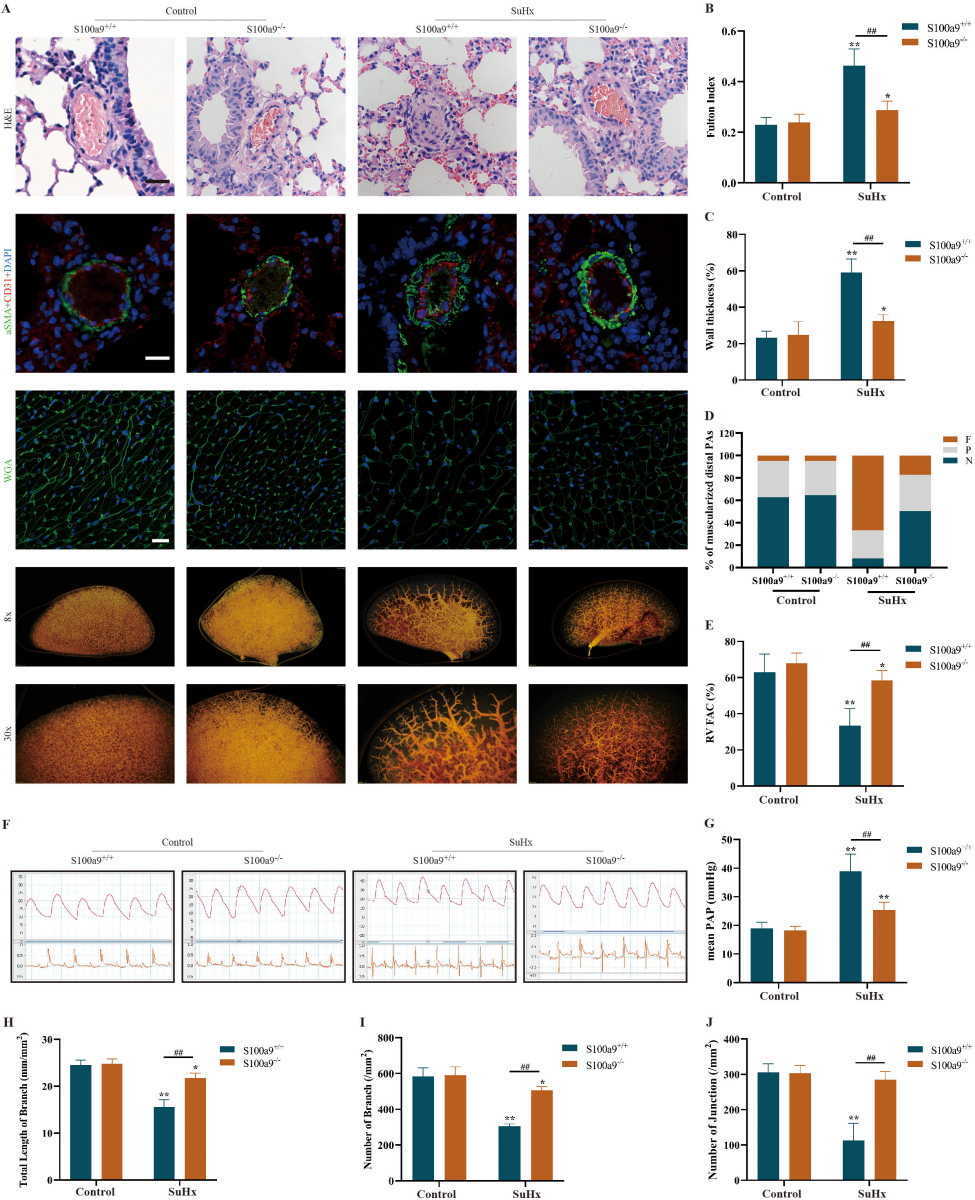
S100a9 in pulmonary vascular remodeling/PAH. **(A)** First line: H & E staining of mouse lung terminal arterioles. Second line: Immunofluorescence staining of aSMA (green), CD31 (red), and DAPI (blue) in mouse lung terminal arterioles. Third line: WGA staining with WGA in green and DAPI in blue. Fourth line: Representative pulmonary angiography images of the lungs at 8×. Fifth line: Representative pulmonary angiography images of the lungs at 30×. **(B)** Fulton index of different groups. **(C)** Vascular wall thickness in mouse lung terminal arterioles. **(D)** Degree of muscularization in mouse pulmonary terminal arterioles. **(E)** The fraction of RV area change was calculated by echocardiography. **(F)** Representative pulmonary hemodynamics images of pulmonary artery pressure measured by a right heart catheter. **(G)** Mean pulmonary artery pressure obtained from right heart catheter measurements in mice. **(H)** The total length of lung branches. **(I)** Number of lung branches. **(J)** Number of mouse connections in the lungs. All data are presented as the mean ± SEM (n=6). *p < 0.05, **p < 0.01 when compare to the normoxia subgroup; ^##^p <0.01 represents the significant difference between S100a9 knockout and wildtype in the SuHx. Scale bar=20 μm.

Integrated scRNA-seq and scATAC-seq analyses identified S100a9 as a DEG in granulocytes, with concomitant upregulation observed in CCR2^+^ proinflammatory macrophages. To determine the contribution of inflammatory cell-derived S100a9 to PAH pathogenesis, we utilized a global S100a9-deficient mouse model. The results showed that the pulmonary vascular wall was significantly thickened, the lumen was reduced or even occluded, and the number and degree of muscular distal pulmonary arteries, Fulton index and cardiomyocyte size were increased in S100a9^+/+^ mice after SuHx exposure. However, S100a9 knockout significantly attenuated these changes (**Figures 9A–D**). The results obtained from right ventricular catheter measurements and echocardiography showed that mean pulmonary artery pressure (mPAP) significantly increased and the fraction of right ventricular area change (RV FAC) decreased significantly in S100a9^+/+^ mice after SuHx exposure, while S100a9 knockout significantly ameliorated these changes (**Figures 9E–G**). The results obtained from pulmonary angiography further confirmed that the total length of branches, number of branches and number of branch connections were decreased in S100a9^+/+^ mice after SuHx exposure, while S100a9 knockout significantly reversed these changes (**Figures 9A, H–J**).

## Discussion

In this study, scRNA-seq and scATAC-seq were applied to construct a lung cell atlas and explore the underlying mechanisms of pulmonary vascular remodeling/PAH. The activation of inflammatory cells and inflammatory signaling pathways was observed during PAH development. Interestingly, the HIF pathway was specifically activated in granulocytes and monocytes/macrophages after SuHx exposure but not in other cells, which may be the basis of the inflammatory mechanisms of PAH. Further analysis highlighted alterations in the characteristics, cell–cell interactions and molecular regulatory mechanisms of key inflammatory cells in the process of PAH. Gene knockout technology suggested a pivotal role for S100a9 as a potential regulator of PAH.

The most significant contribution of this study is the identification of the basis of the inflammatory mechanisms of PAH. Initially, the results of our study emphasize that hypoxia-induced EC injuries may lead to barrier disruption and subsequent infiltration of inflammatory cells. Furthermore, the study reveals that the HIF pathway is specifically activated in granulocytes and monocytes/macrophages and not in other cells after SuHx exposure, which suggests that granulocyte- and monocyte/macrophage-mediated inflammation may be the core mechanism during the development of PAH. Based on scRNA-seq, previous studies have reported that extensive activation of the nuclear factor kappa-B (NFκB) signaling pathway and inhibition of the interferon signaling pathway may play a key role in SuHx and MCT models of PH, suggesting that inflammatory activation plays a critical role in the occurrence of PH (5). It has been reported that inflammatory activation is a key event in the occurrence and development of various types of PH (25), including neutrophil aggregation, monocyte/macrophage activation, lymphocyte activation, and DC participation. Moreover, a previous study conducted scRNA-seq specifically for immune cells in patients with PAH and found that γδT lymphocytes and pDCs are two key cell subsets of patients with idiopathic PH (4). Moreover, a recent study reported that specific monocyte subsets and HIF-1α downregulation might play a vital role in the pathogenesis of high-altitude pulmonary hypertension (HAPH) based on scRNA-seq analysis of the peripheral blood mononuclear cells of patients with HAPH (26). The infiltration of neutrophils and macrophages is one of the consistent pathological features of PH induced by hypobaric hypoxia (such as high-altitude hypoxia) (27).

In the present study, we found that the proportion of neutrophils in the lung tissue of mice was significantly increased after SuHx exposure, which may be partly due to the fact that inflammatory factors (such as IL-6) can induce the mobilization of neutrophils from bone marrow during pulmonary vascular remodeling (28). Subsequently, activated neutrophils release neutrophil extracellular traps (NETs), which are reticular structures consisting of DNA histones and proteins and play a dominant role in the neutrophil-mediated innate immune response. In PH patients, many neutrophils producing NETs and extensive NETosis (an inflammatory cell death mode of neutrophils) were found. NETs induce NFκB-dependent endothelial angiogenesis and the release of endothelin-1 from human pulmonary ECs, promote the proliferation of pulmonary SMCs, and ultimately lead to pulmonary vascular remodeling (29). Furthermore, clinical studies have found that the ratio of neutrophils to lymphocytes is closely related to the severity and clinical outcomes of PH patients (30, 31), indicating that neutrophils play an important role in the occurrence and development of PH.

The role of macrophage activation in the development of PH is well understood. The accumulation and presence of macrophages is an important feature of PH and plays a key role in pulmonary artery remodeling through various cellular and molecular mechanisms, involving factors including CCL2 and CX3CL1 chemokines, adventitial fibroblasts, glucocorticoid-regulated kinase 1, leukotriene B4, bone morphogenetic protein receptor type 2 (BMPR2), macrophage migration inhibitory factor, and platelet reaction-protein-1 (32). Macrophage activation could lead to T lymphocyte activation and induce the production of IL-1, IL-6, IL-10, TNF-α and other inflammatory factors, thus promoting inflammatory cell infiltration (33). Among these inflammatory factors, IL-6 could promote the upregulation of VEGF, induce the downregulation of BMPR2 and TGFβR, and promote the proliferation of SMCs and exert an anti-apoptosis effect on these cells (34). In addition, the inflammatory activation of macrophages could promote the expression of IL-18 and IL-1 by activating NLRP3, thus stimulating the proliferation of PASMCs and leading to pulmonary vascular remodeling (35). It has been shown that IL-1β-mediated PASMC proliferation can be inhibited by IL-1R1 knockout (36), and IL-1 antagonists can effectively reduce vascular inflammation and significantly reduce pulmonary vascular remodeling (37). Consistent with our study, previous results from scRNA-seq showed that intermittent hypoxia exposure resulted in an increase in M1-type macrophages and a decrease in M2-type macrophages in a mouse model, suggesting that macrophage polarization may be an important mechanism of PAH occurrence (38).

S100a8/a9, also known as calprotectin, is a calcium-containing protein derived from neutrophils and monocytes/macrophages that regulates neutrophil and macrophage migration and chemokine and inflammatory cytokine expression (39). In this study, it was found that the expression of S100a9 in macrophages was increased significantly after SuHx exposure. Previous studies have shown that the increased expression of S100a9 in macrophages can lead to the formation of reactive oxygen species-dependent NETs, which can in turn activate macrophages to form a positive feedback loop of inflammatory responses (40). The overexpression of S100a9 inhibits macrophage differentiation through the TLR4-NFkB signaling pathway, leading to increased inflammatory responses (41). In acute kidney injury, S100a8/a9^+^ macrophages undergo infiltration, and blocking S100a8/a9 can reduce kidney inflammation, alleviate kidney injury and fibrosis, improve kidney function and improve the survival rate (42). Moreover, S100a9 is one of the ligands of the receptor for advanced glycation end products (RAGE). Studies have reported that serum RAGE levels are significantly increased in patients with chronic thromboembolic PH and idiopathic PH, suggesting that the RAGE signaling pathway may be involved in the development of PH (43). Accordingly, in this study, the knockout of S100a9 prevented pulmonary vascular remodeling, the elevation of pulmonary pressure and right heart hypertrophy, suggesting that S100a9 is a key regulatory factor in the occurrence and development of PAH. In addition, neutrophil S100a9 interacts with C/EBPβ to synergistically activate the promoters of prostaglandin E2 and enhance its production, thereby contributing to M2 polarization in macrophages (44). Based on this inference, the knockout of S100a9 may cut off intercellular communication between neutrophils and macrophages, thus alleviating pulmonary vascular remodeling. Consequently, the multifaceted involvement of S100a9 in the inflammatory and vascular processes underpinning PAH progression.

Through ATAC sequencing, we found that the expression levels of FosL1, JunB, Id1 and JunD were significantly increased after SuHx exposure. FosL1 is a transcriptional regulator of activator protein 1 (AP-1) and mediates the inflammatory responses of macrophages (45). The AP-1 complex proteins c-Fos and c-Jun are involved in pulmonary vascular SMC proliferation (46), while the inhibition of meprin β inhibits pulmonary vascular SMC proliferation and pulmonary vascular remodeling by silencing AP-1 expression. Moreover, the ACE inhibitor enalapril attenuates pulmonary hypertension by inhibiting the TNF-mediated activation of NFκB and AP-1 (47). We also found that the expression of PPARγ and PPARα decreased significantly after SuHx exposure. It has been reported that the PPARγ agonist rosiglitazone can reverse pulmonary vascular remodeling, the elevation of pulmonary artery pressure, and right heart failure by regulating fatty acid oxidation (48). Id1 is also a proinflammatory factor that promotes macrophage activation by regulating inflammatory signaling pathways such as NF-κB (49). These results further confirm the key role of inflammatory cells and inflammatory signaling pathways in the occurrence and development of PAH and may provide a new basis for targeted drug development.

### Limitations

This study has several limitations that merit further discussion. First, our current findings are primarily based on bioinformatic analyses, which suggest a potential regulatory link. The regulation of the screened transcription factors on S100a9 in CCR2^+^ macrophages was not substantial for the lack of validation with ChIP-qPCR or other techniques. Second, although global S100a9 knockout mice effectively capture the combined contribution of the two major inflammatory cell populations, this approach does not allow for the exclusion of contributions from other cell types, as the knockout is not cell−type specific.

## Conclusion

We constructed a single-cell transcriptome atlas and a single-cell chromatin accessibility atlas for an PAH model. Our findings suggested that S100a9, identified as a DEG in granulocytes and concurrently upregulated in CCR2^+^ proinflammatory macrophages, might be associated with the development of PAH through its role in mediating inflammatory cell activation. These findings provide new insights into PAH pathogenesis, yet the underlying mechanisms await further investigation.

## Data Availability

The datasets presented in this study can be found in online repositories. The names of the repository/repositories and accession number(s) can be found in the article/**Supplementary Material**.
