# Supplementary material for: Integrative single-cell RNA-seq and ATAC-seq analysis reveals the key role of inflammatory cell activation in pulmonary arterial hypertension
Source: Front Immunol. 2026 Apr 15;17:1796116. doi: 10.3389/fimmu.2026.1796116 (PMC13124965; doi:10.3389/fimmu.2026.1796116)
Supplement: Supplementary file 1 [file DataSheet1.docx]

**Supplemental figures**

**Supplemental figure 1**

**
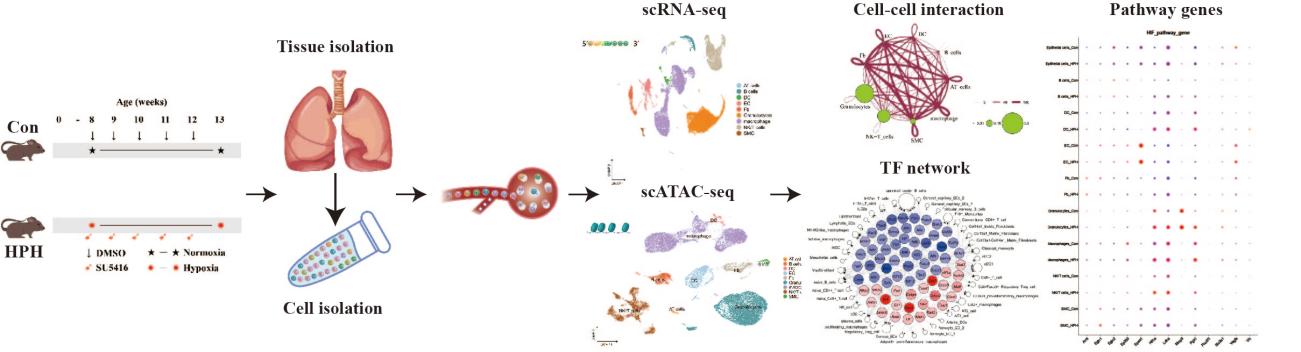
**

**Supplemental figure 1. Overview of the study design.** Construction of the HPH mouse model and Con mouse model (left). Cell suspensions of lung tissue from HPH group (n=4) and Con group (n=4) were subjected to scRNA-seq and scATAC-seq (middle), and a single-cell transcriptomic atlas and single-cell chromatin accessibility atlas were constructed. Moreover, cell type identification, cell‒cell interaction analysis, transcription factor identification and pathway genes analysis were performed (right).

**Supplemental figure 2**


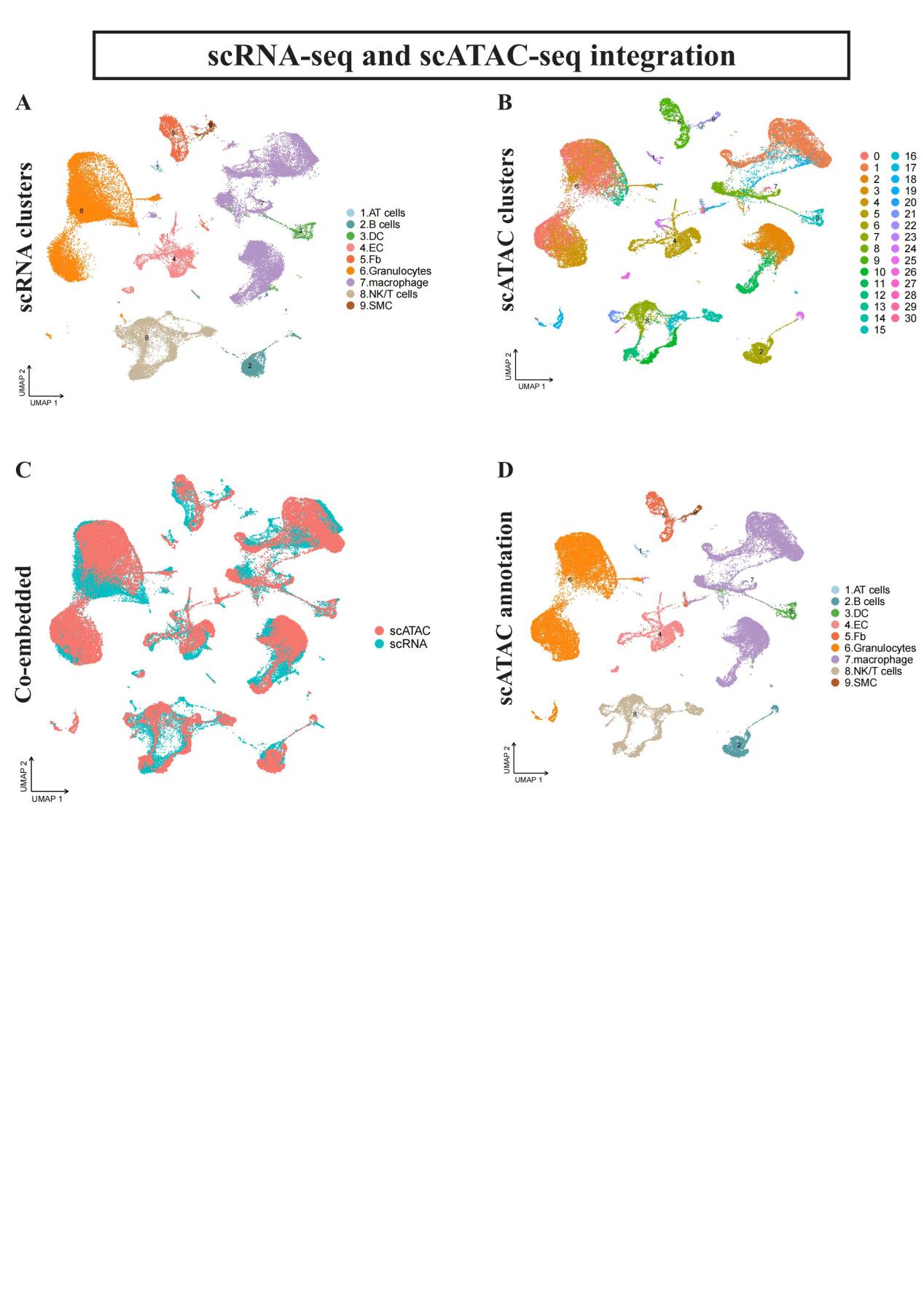


**Supplemental figure 2. Integration of scRNA-Seq and scATAC-Seq Datasets. (A)** UMAP representation of scRNA-seq datasets. **(B)** UMAP representation of scATAC-seq datasets. **(C)** UMAP representation of the co-embedded scATAC-seq (red) and scRNA-seq (blue) datasets. **(D)** Annotations used for the scRNA-seq shown in Figure 1B were used and superimposed onto each cell cluster.

**Supplemental figure 3**

**
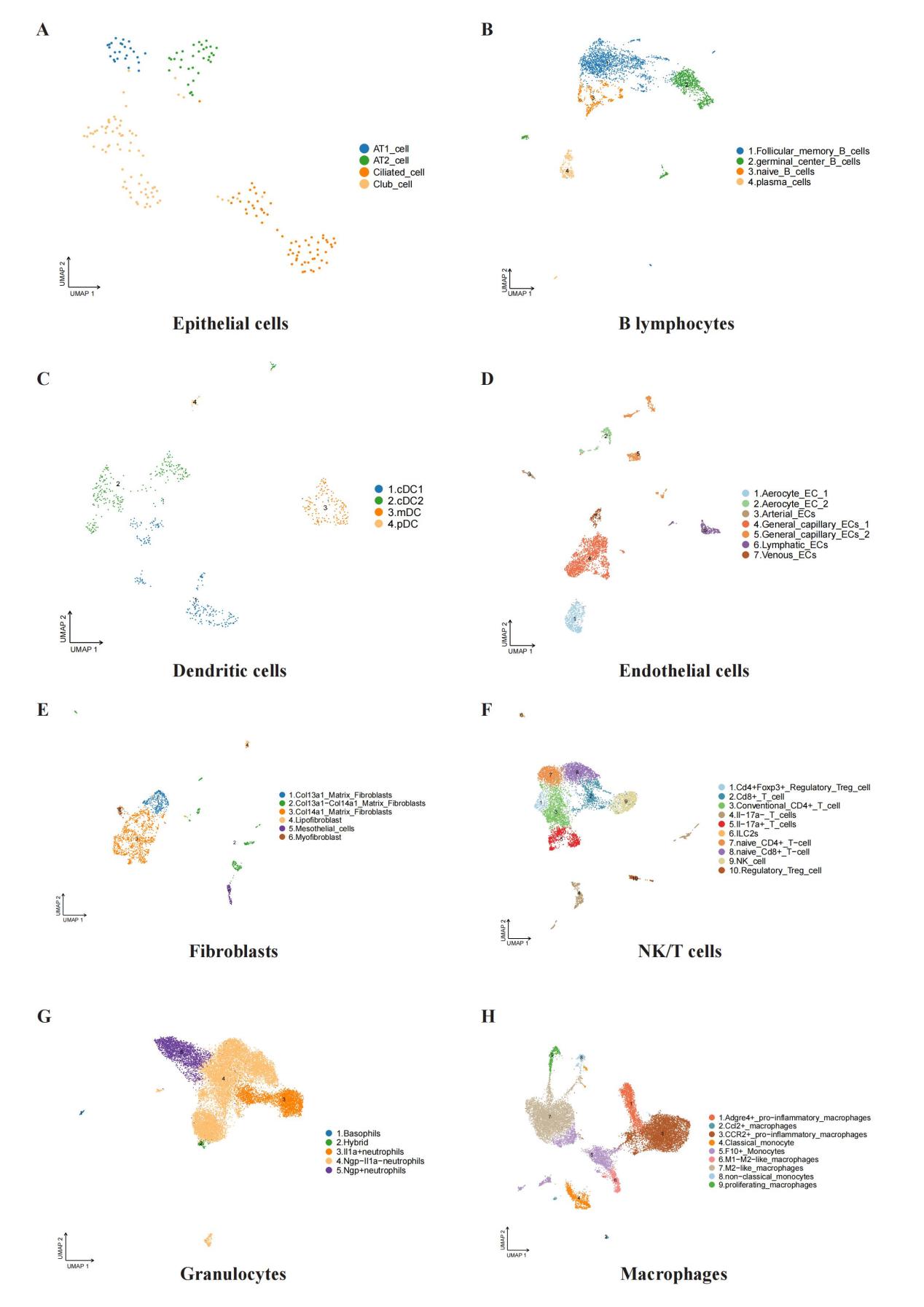
**

**Supplemental figure 3. UMAP representations of different subgroup cells.** Single-cell transcriptomic atlas of **(A)** epithelial cells, **(B)** B lymphocytes, **(C)** dendritic cells, **(D)** endothelial cells, **(E)** fibroblasts, **(F)** NK/T cells, **(G)** granulocytes and **(H)** macrophages.

**Supplemental figure 4**

**
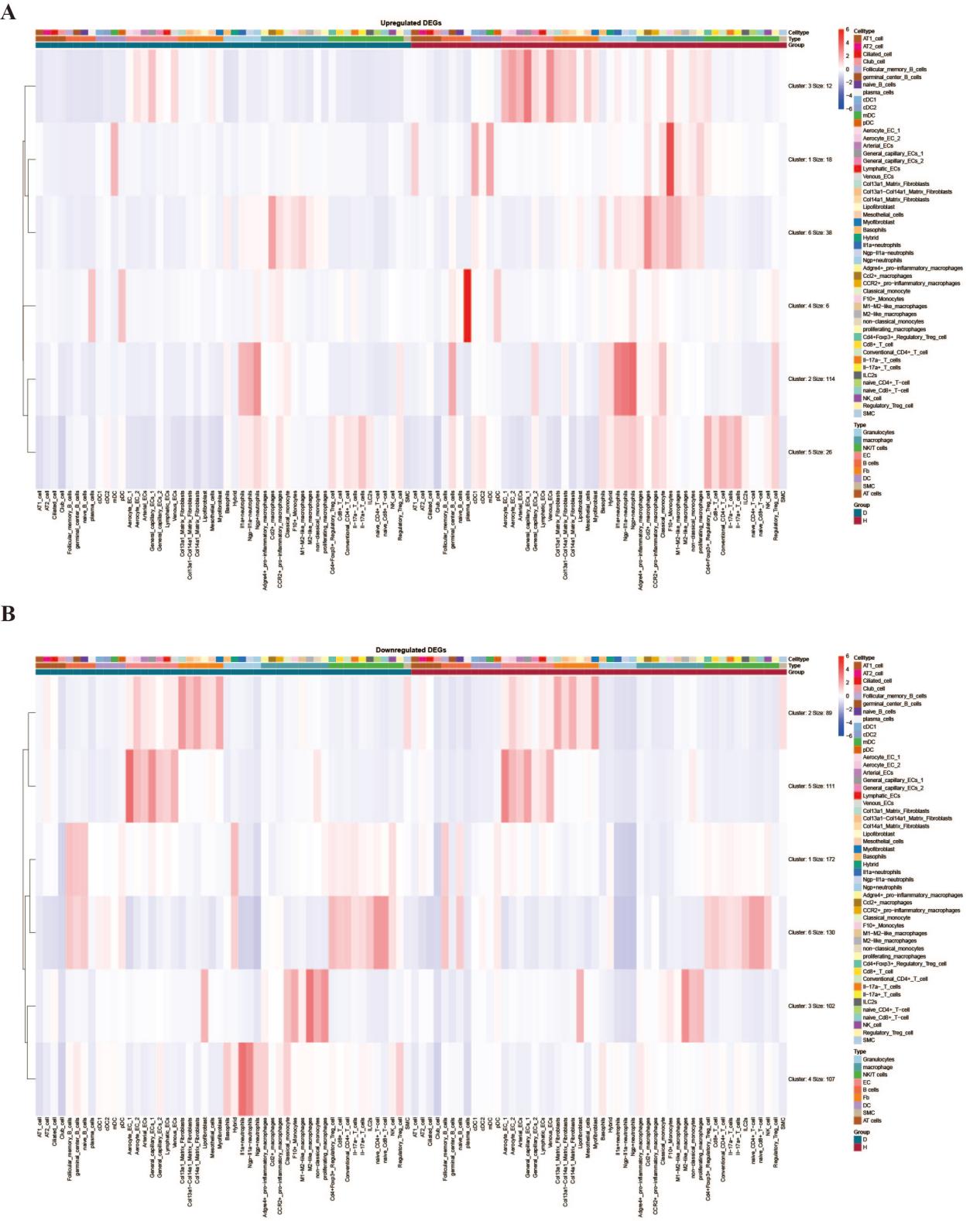
**

**Supplemental figure 4. (A)** Heatmap showing 6 upregulated clusters of DEGs across different cell types in the lungs of Con and HPH group mice. **(B)** Heatmap showing 6 downregulated clusters of DEGs across different cell types in the lungs of Con and HPH groups.

**Supplemental figure 5**

**
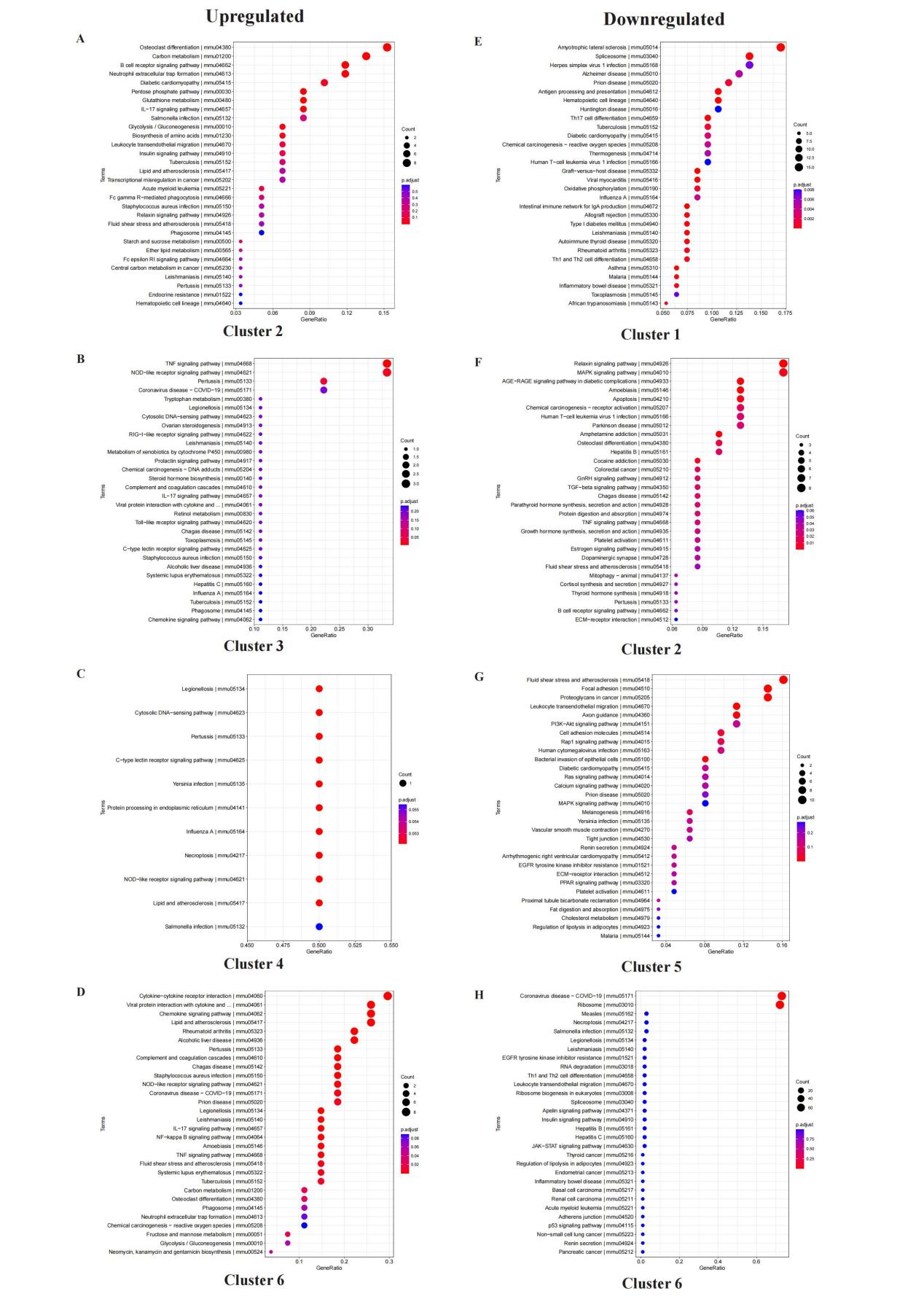
**

**Supplemental figure 5. KEGG enrichment analysis of differential gene expression in different clusters.** KEGG enrichment analysis in upregulated **(A)** cluster 2, **(B)** cluster 3, **(C)** cluster 4 and **(D)** cluster 6. And downregulated **(E)** cluster 1, **(F)** cluster 2, **(G)** cluster 5 and **(H)** cluster 6.

**Supplemental figure 6**

**
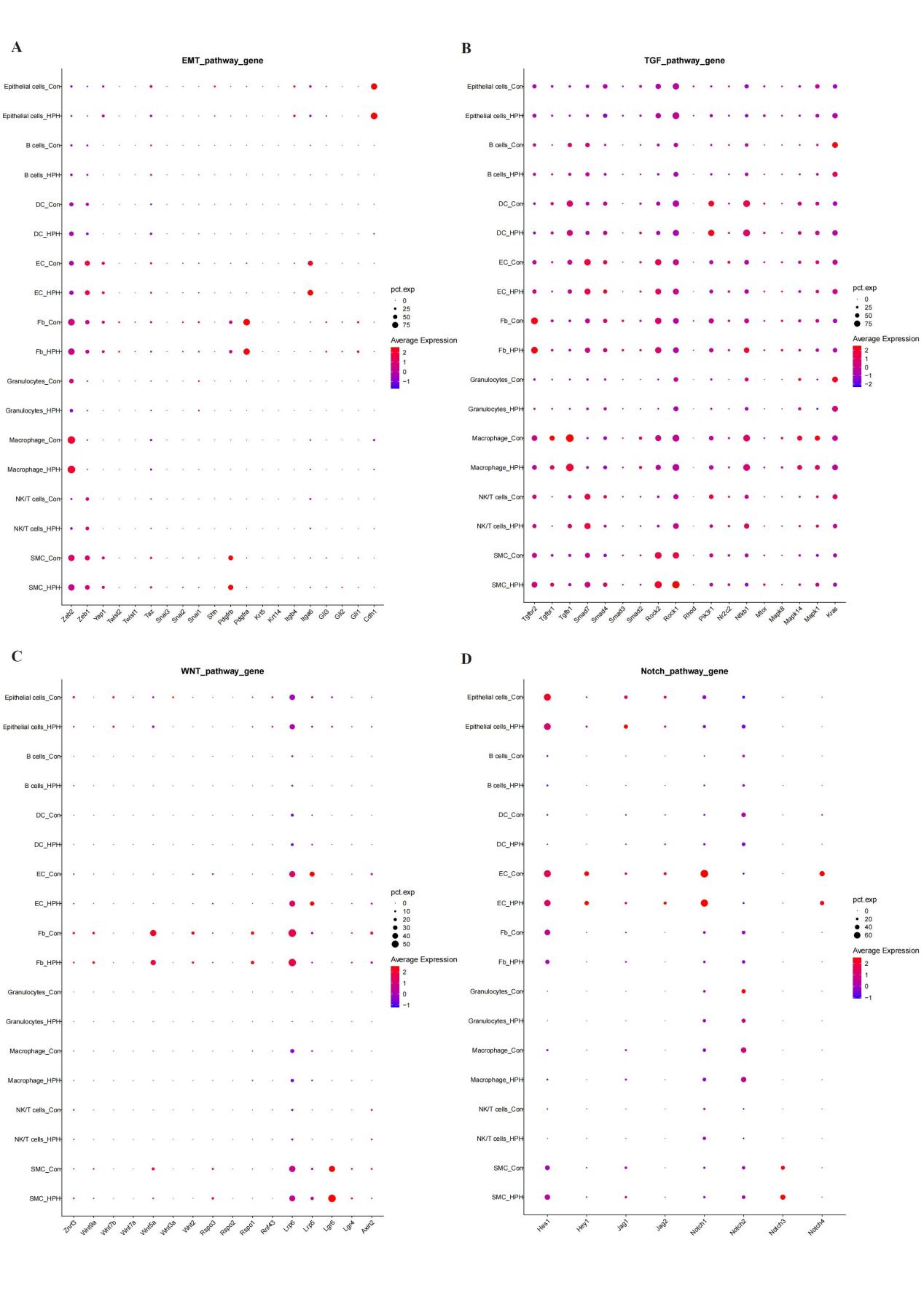
**

**Supplemental figure 6. Pathway genes in nine subgroups of cells in the lung tissues of the Con group and HPH group. (A)** EMT pathway gene, **(B)** TGF pathway gene, **(C)** WNT pathway gene and **(D)** Notch pathway gene.

**Supplemental figure 7**

**
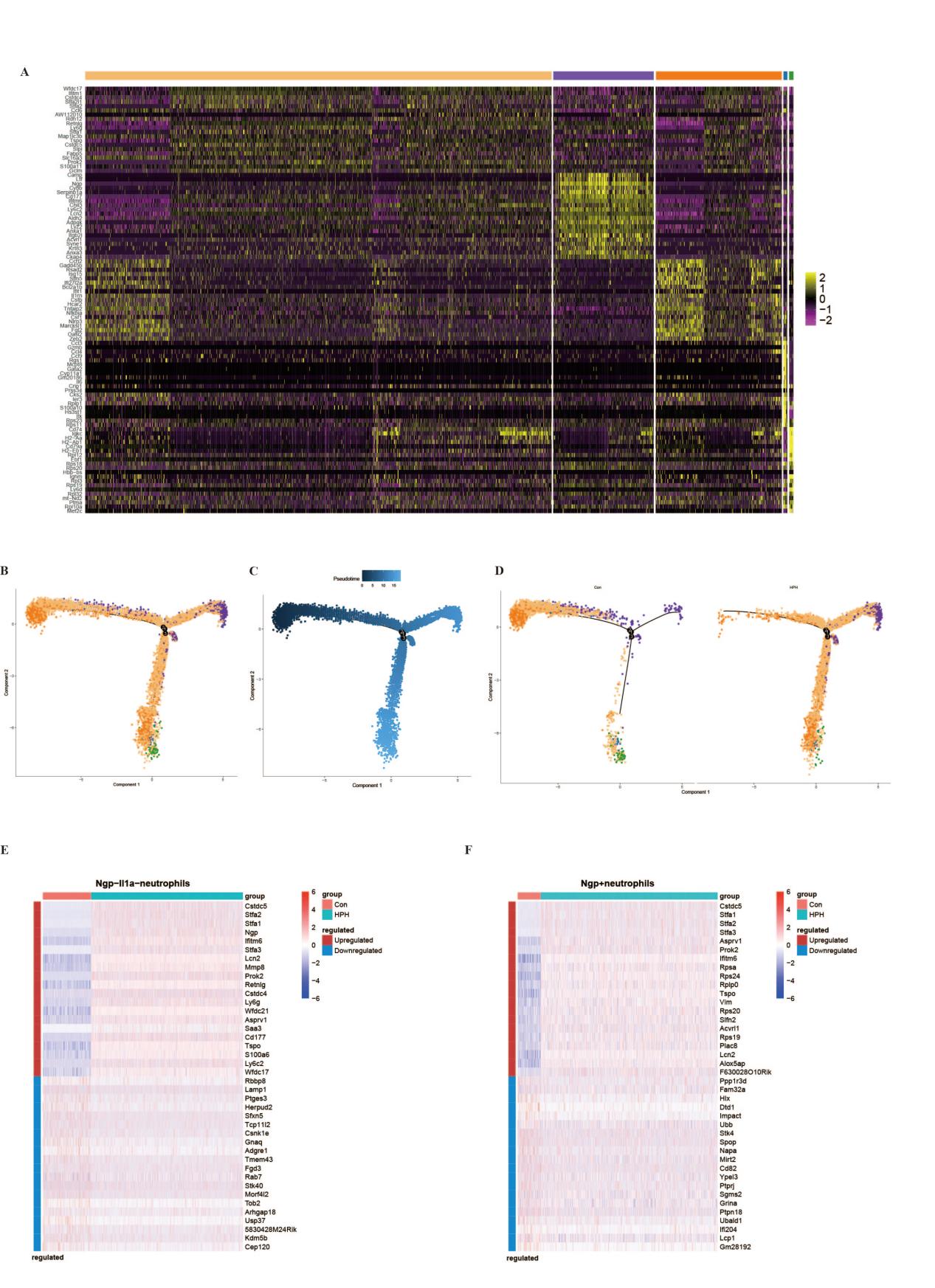
**

**Supplemental figure 7. (A)** Heatmap of the top 20 marker genes corresponding to each cell type in the mouse lung tissue, where each color represents one cell type, and each row represents one gene; color plot as in Figure 5A. **(B)** Pseudotime trajectory analysis of the distribution of granulocyte subgroups in mouse lung tissue, where each color represents one cell type. **(C)** The state of pseudotime trajectory analysis of the distribution of granulocyte subgroups in mouse lung tissue. **(D)** Pseudotime trajectory analysis of the distribution of granulocyte subgroups in the Con group (left) and HPH group (right). **(E)** Heatmap showing the genes that were differentially expressed in Ngp^-^IL1α^-^ neutrophils. **(F)** Heatmap showing the genes that were differentially expressed in Ngp^+^ neutrophils.

**Supplemental figure 8**

**
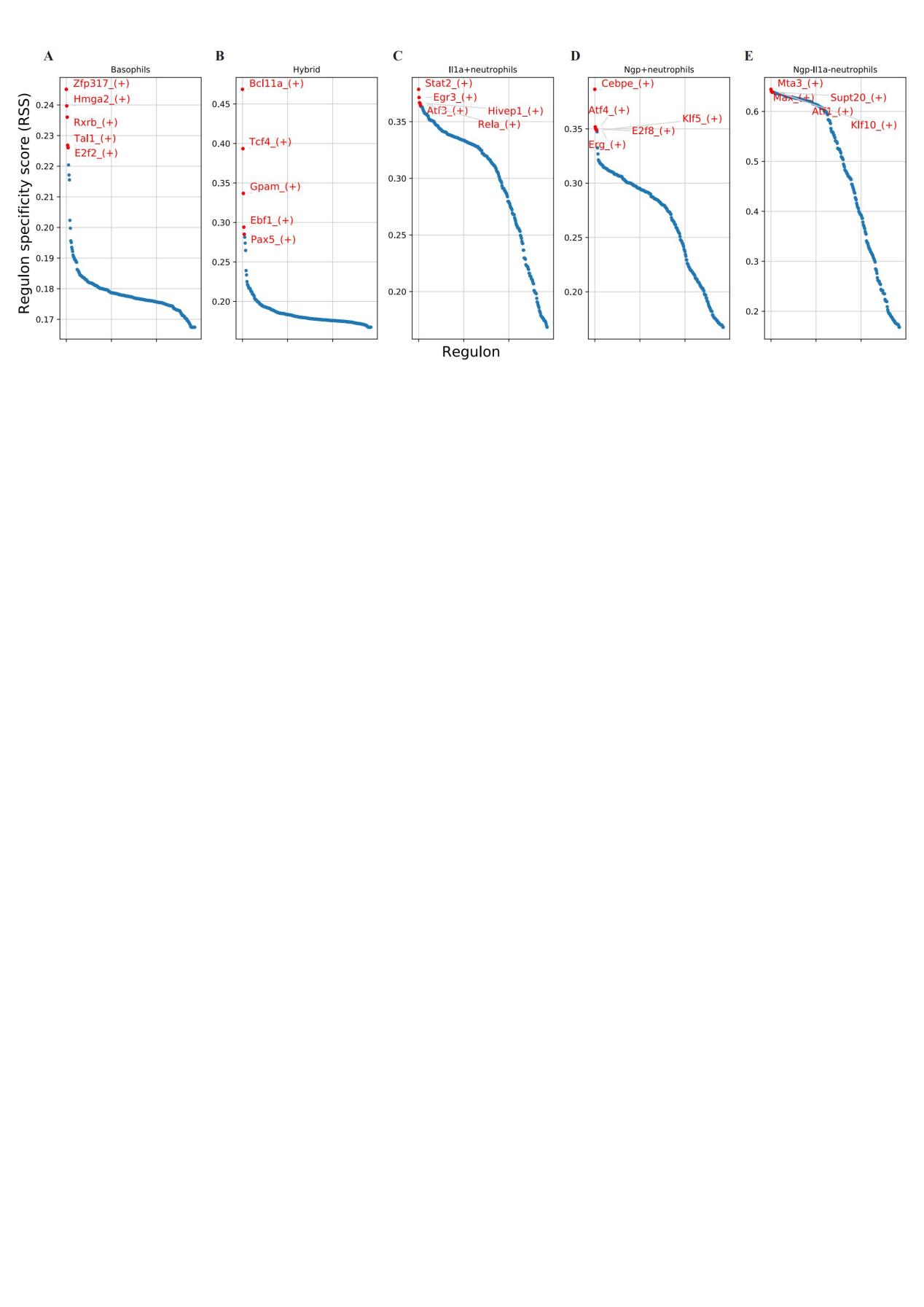
**

**Supplemental figure 8. The top 5 transcriptional factors in each granulocyte subtype by regulon specificity score.** **(A)** Basophils, **(B)** hybrid, **(C)** IL1a^+^ neutrophils, **(D)** Ngp^+^ neutrophils and **(E)** Ngp^-^IL1a^-^ neutrophils.

**Supplemental figure 9**

**
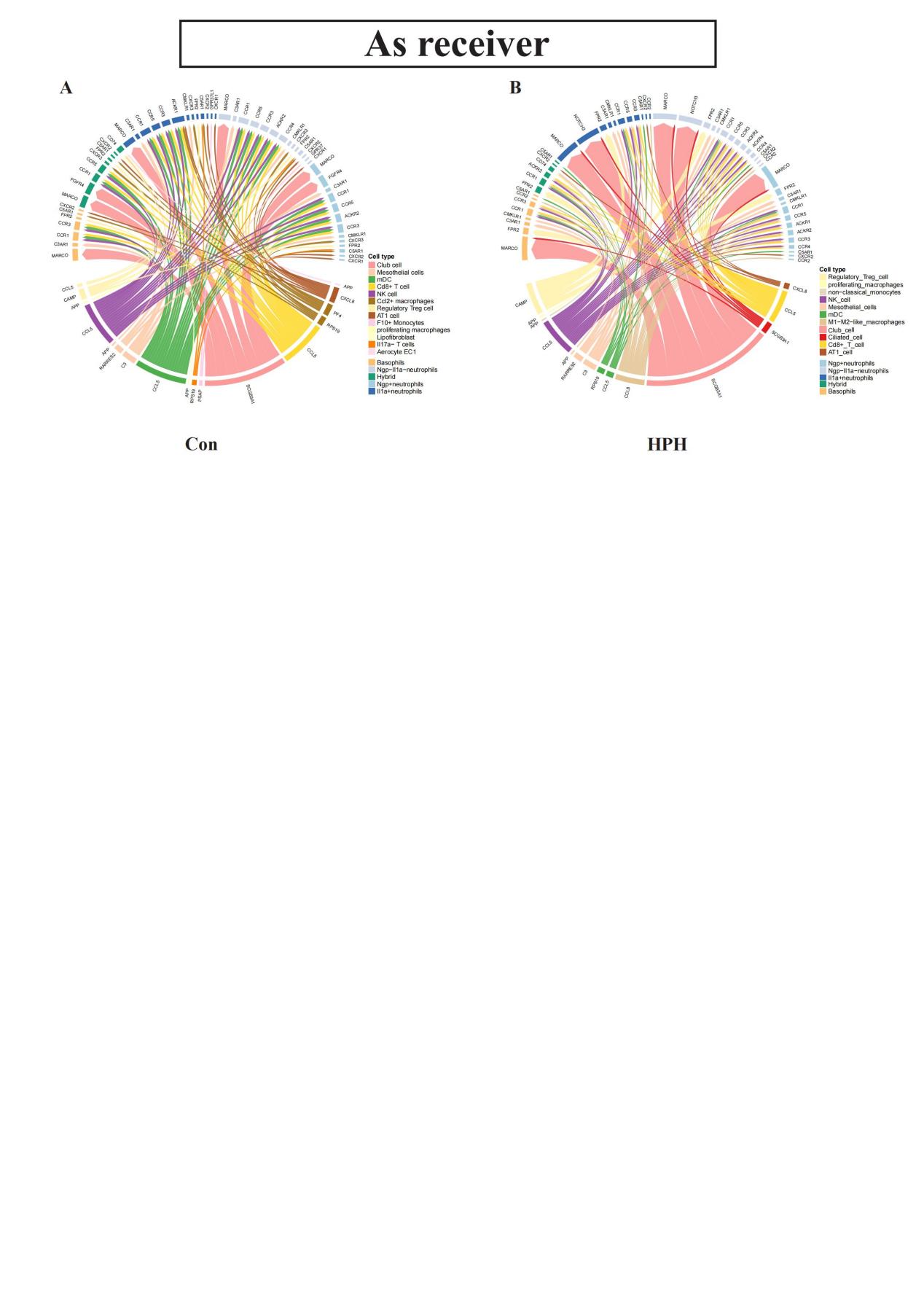
**

**Supplemental figure 9. Circos analyses establish granulocyte-centered communication.** Granulocytic as the receiver in Con group **(A)** and HPH group **(B)**, with the edge thickness is proportional to the edge weight. The edge color marks the source cell type.

**Supplemental figure 10**

**
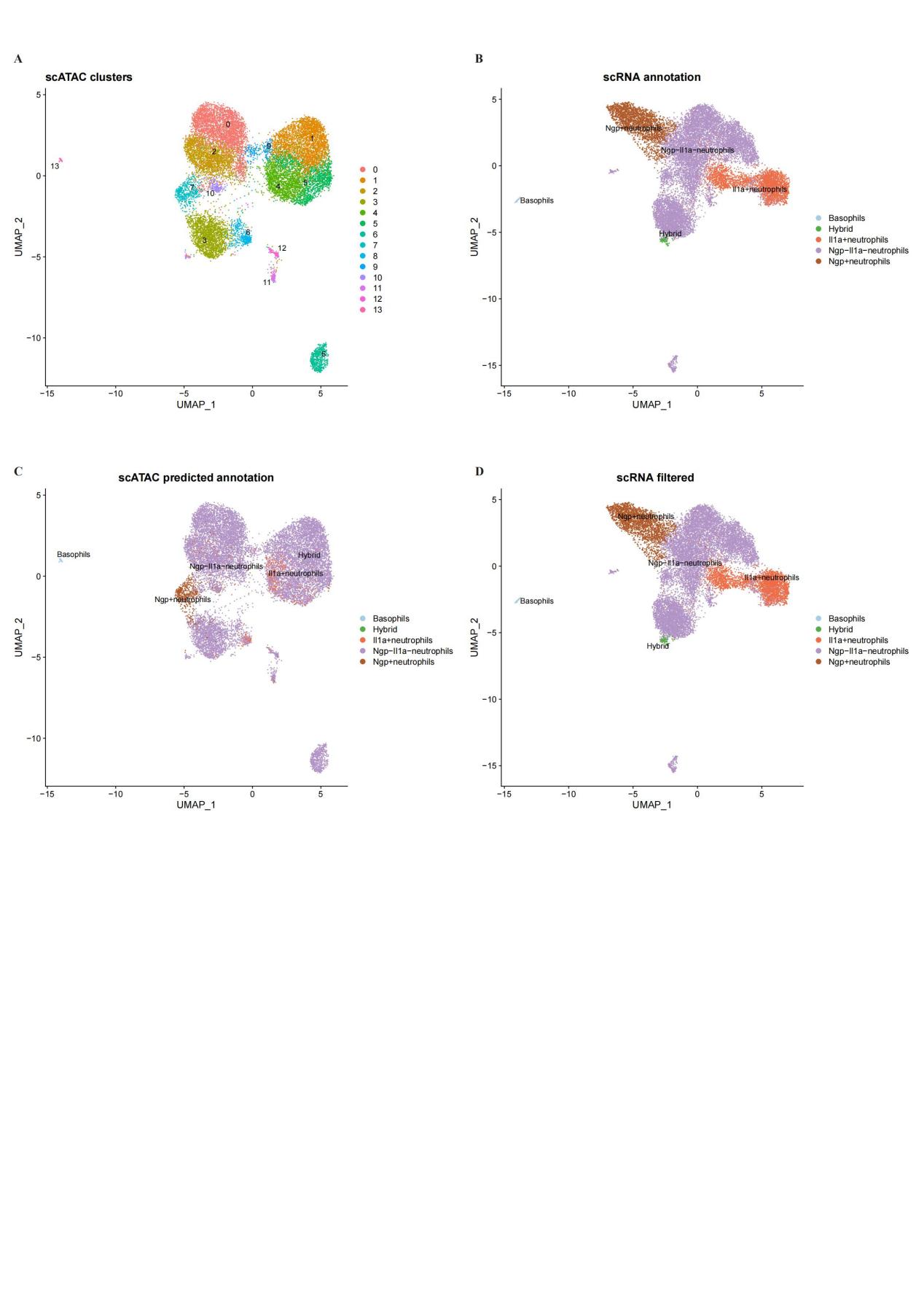
**

**Supplemental figure 10. Integration of scRNA-Seq and scATAC-Seq Datasets in granulocytes. (A)** UMAP representation of scATAC-seq datasets in granulocytes. **(B)** UMAP representation of scRNA-seq datasets in granulocytes. **(C)** UMAP representation of scATAC predicted annotation in granulocytes. **(D)** UMAP representation of scRNA filtered datasets in granulocytes.

**Supplemental figure 11**

**
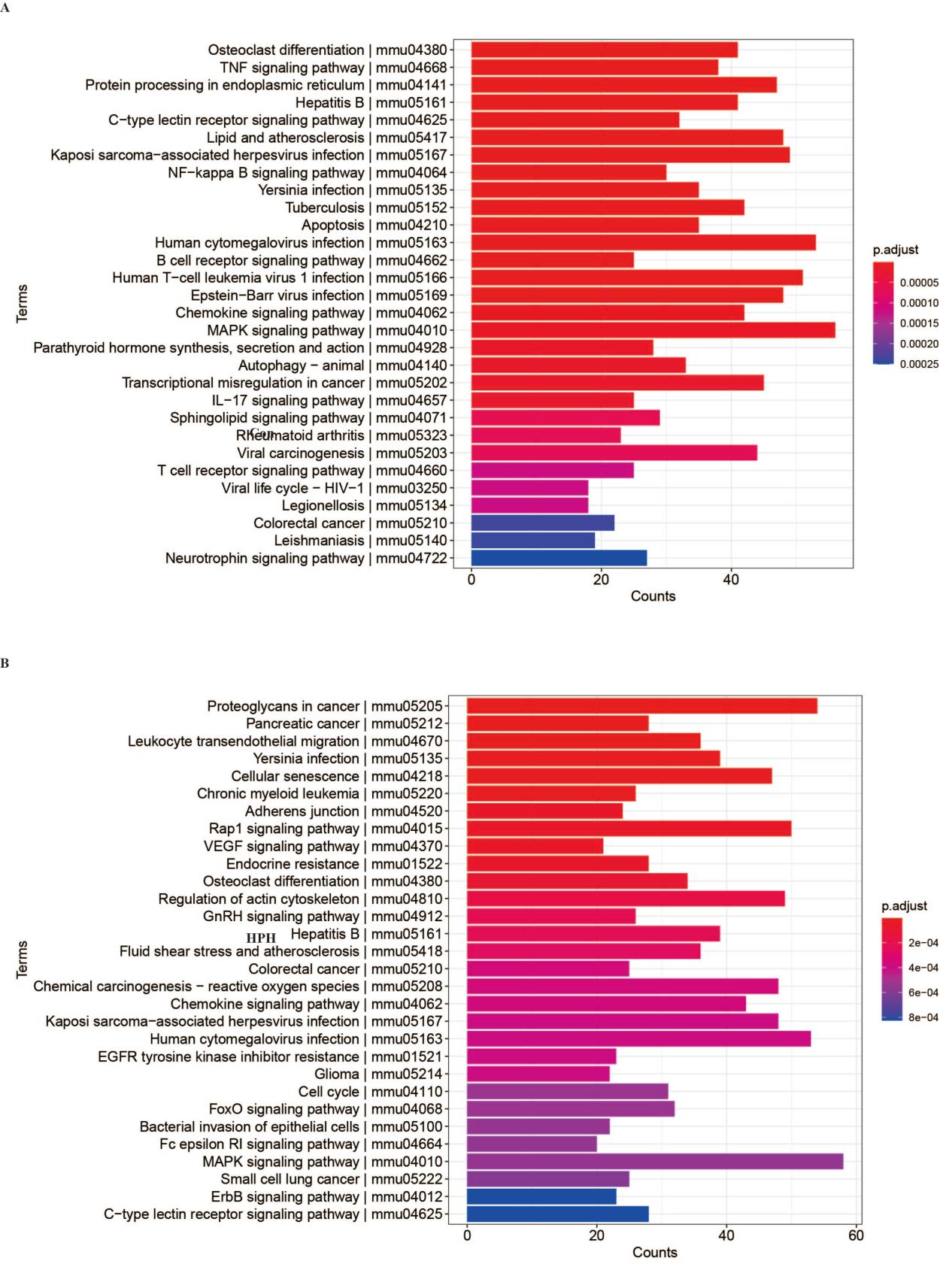
**

**Supplemental figure 11.** KEGG pathways associated with more accessible enhancer regions in lung tissue granulocytes of **(A)** Con and **(B)** HPH group.

**Supplemental figure 12**

**
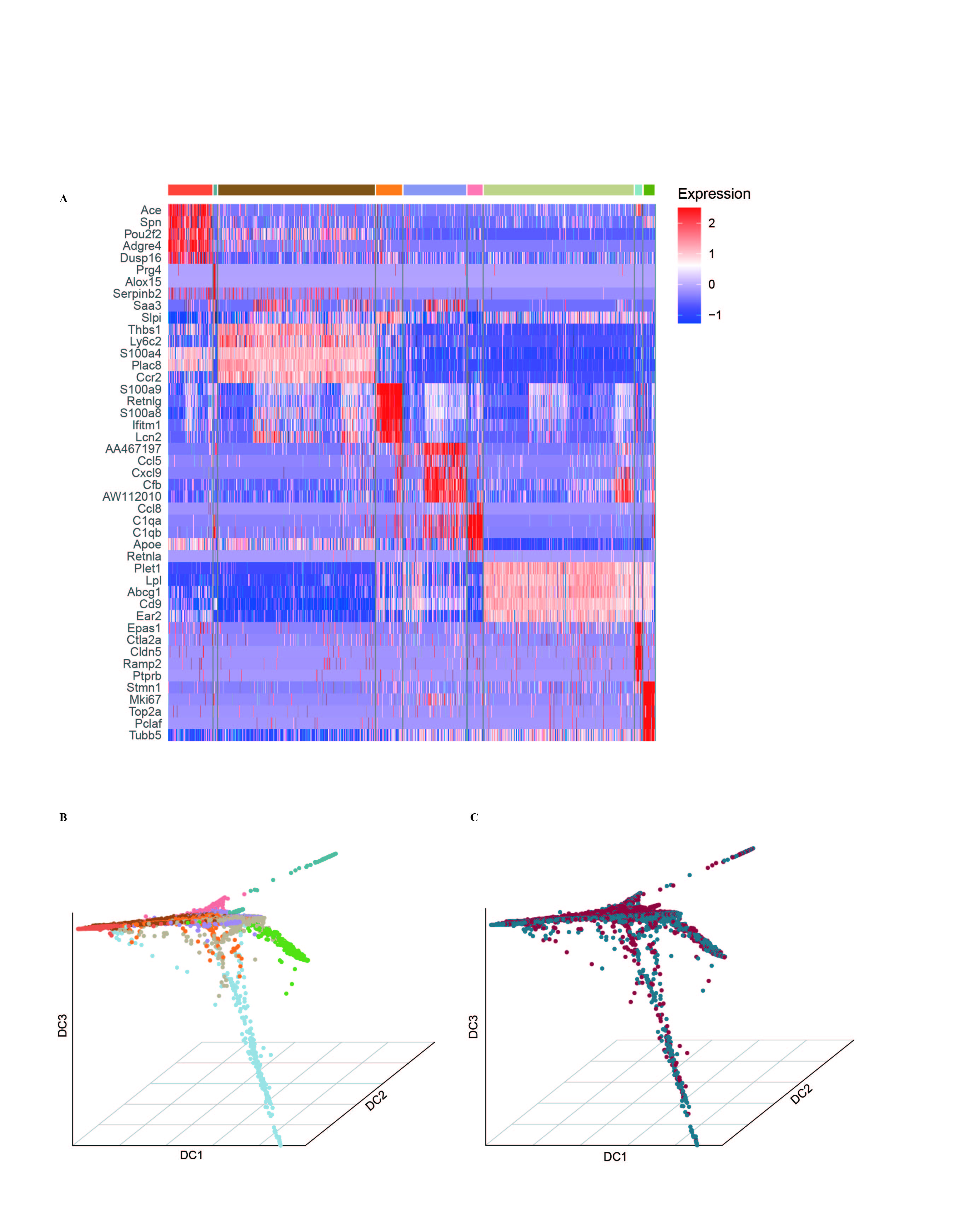
**

**Supplemental figure 12. (A)** Heatmap of the top 5 marker genes corresponding to each cell type in the mouse lung tissue, where each color represents one cell type, and each row represents the expression of one gene; color plot as in Figure 7A. **(B)** Pseudotime trajectory analysis of the distribution of monocytes/macrophages in mouse lung tissue, where each color represents one cell type. **(C)** Pseudotime trajectory analysis of the distribution of monocytes/macrophages in the Con group (blue) and HPH group (red).

**Supplemental figure 13**

**
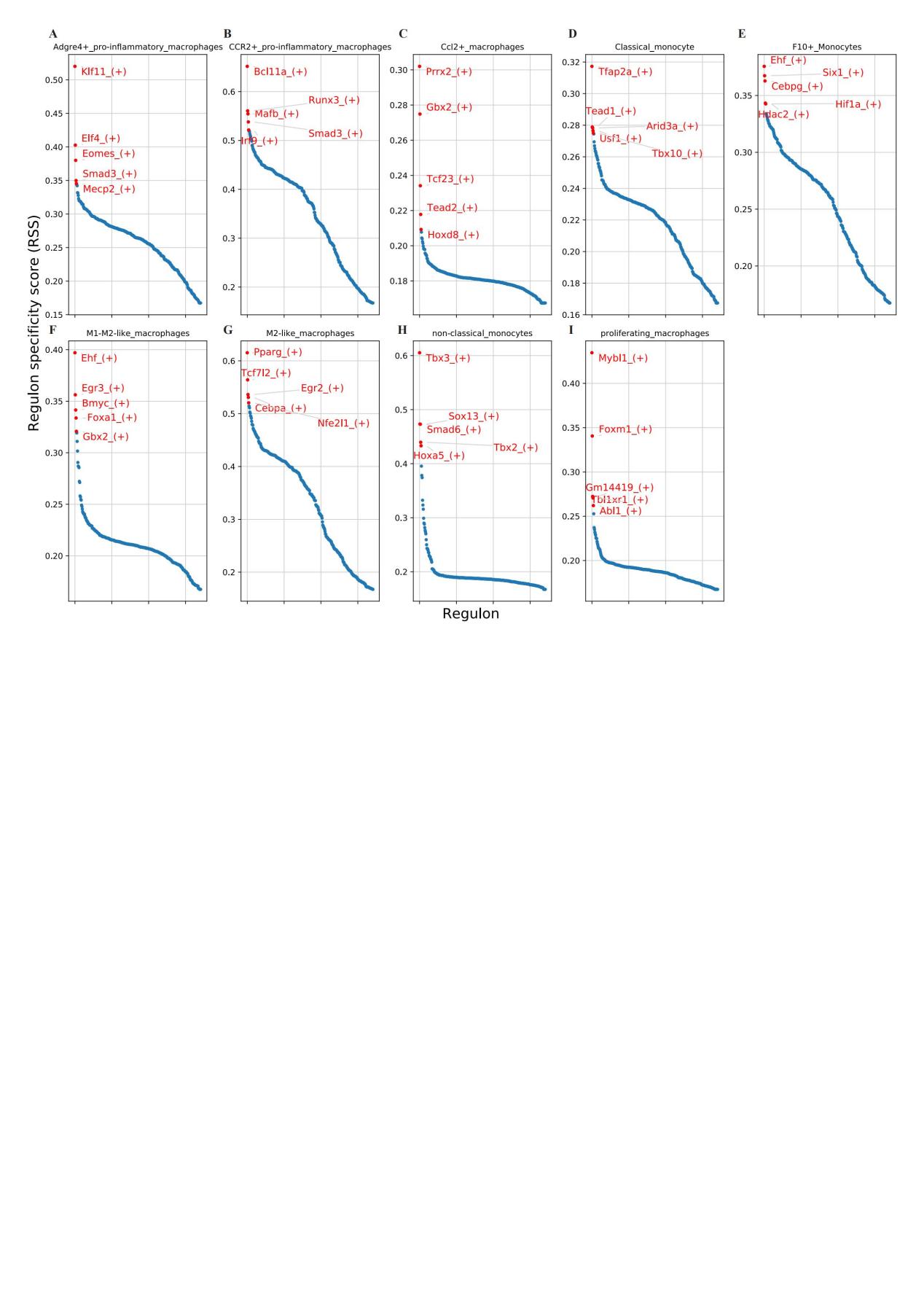
**

**Supplemental figure 13. The top 5 transcriptional factors in each macrophage subtype by regulon specificity score.** **(A)** Adgre4^+^ pro-inflammatory macrophagess, **(B)** CCR2^+^ pro-inflammatory macrophages, **(C)** Ccl2^+^ macrophages, **(D)** classical monocyte, **(E)** F10^+^ Monocytes, **(F)** M1-M2 like macrophages, **(G)** M2-like macrophages, **(H)** non-classical monocytes and **(I)** proliferating macrophages.

**Supplemental figure 14**

**
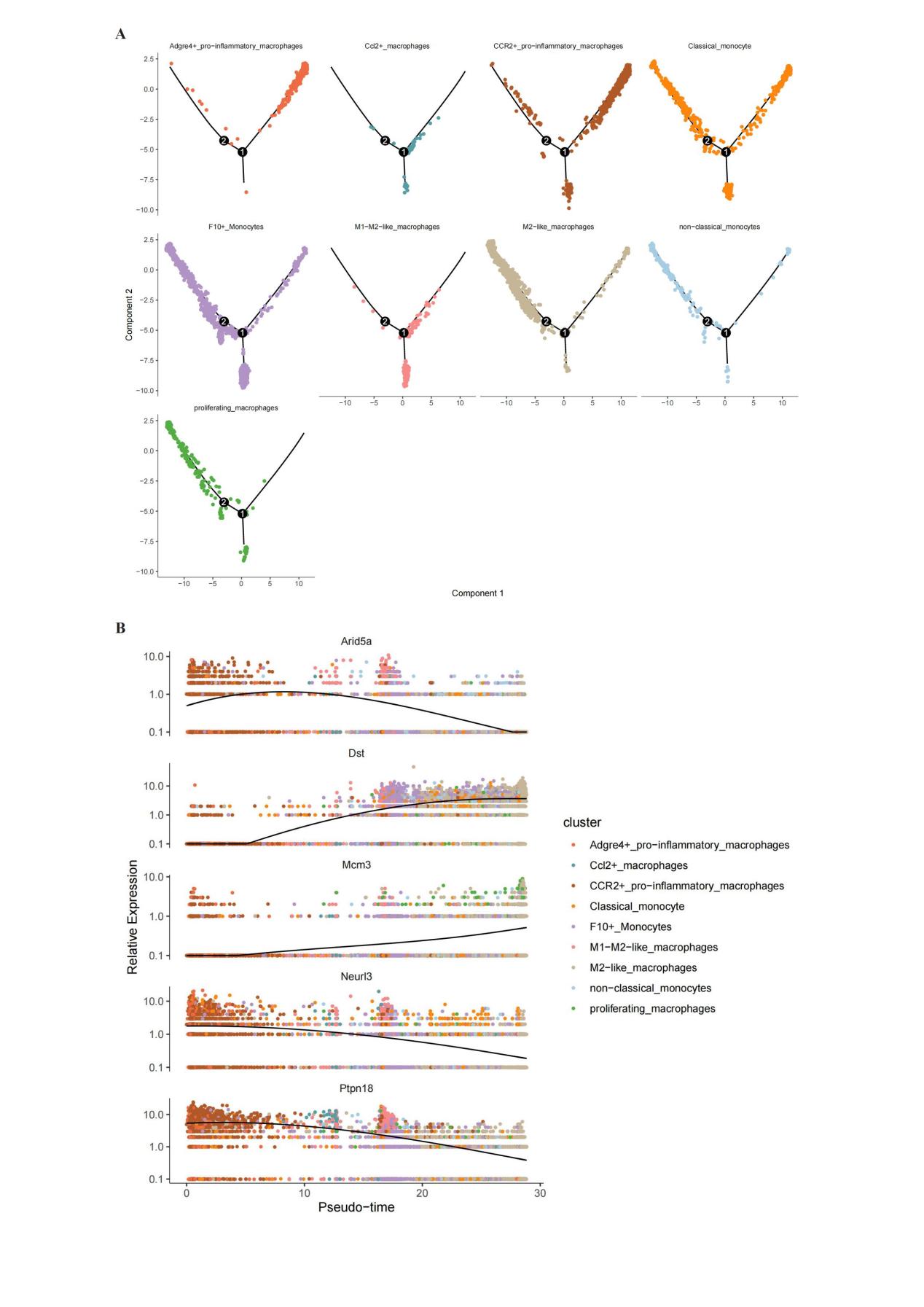
**

**Supplemental figure 14. (A)** Pseudotime trajectory analysis of the distribution of macrophage subgroups in mouse lung tissue. **(B)** Different top gene express in macrophage subgroups among pseudotime.

**Supplemental figure 15**

**
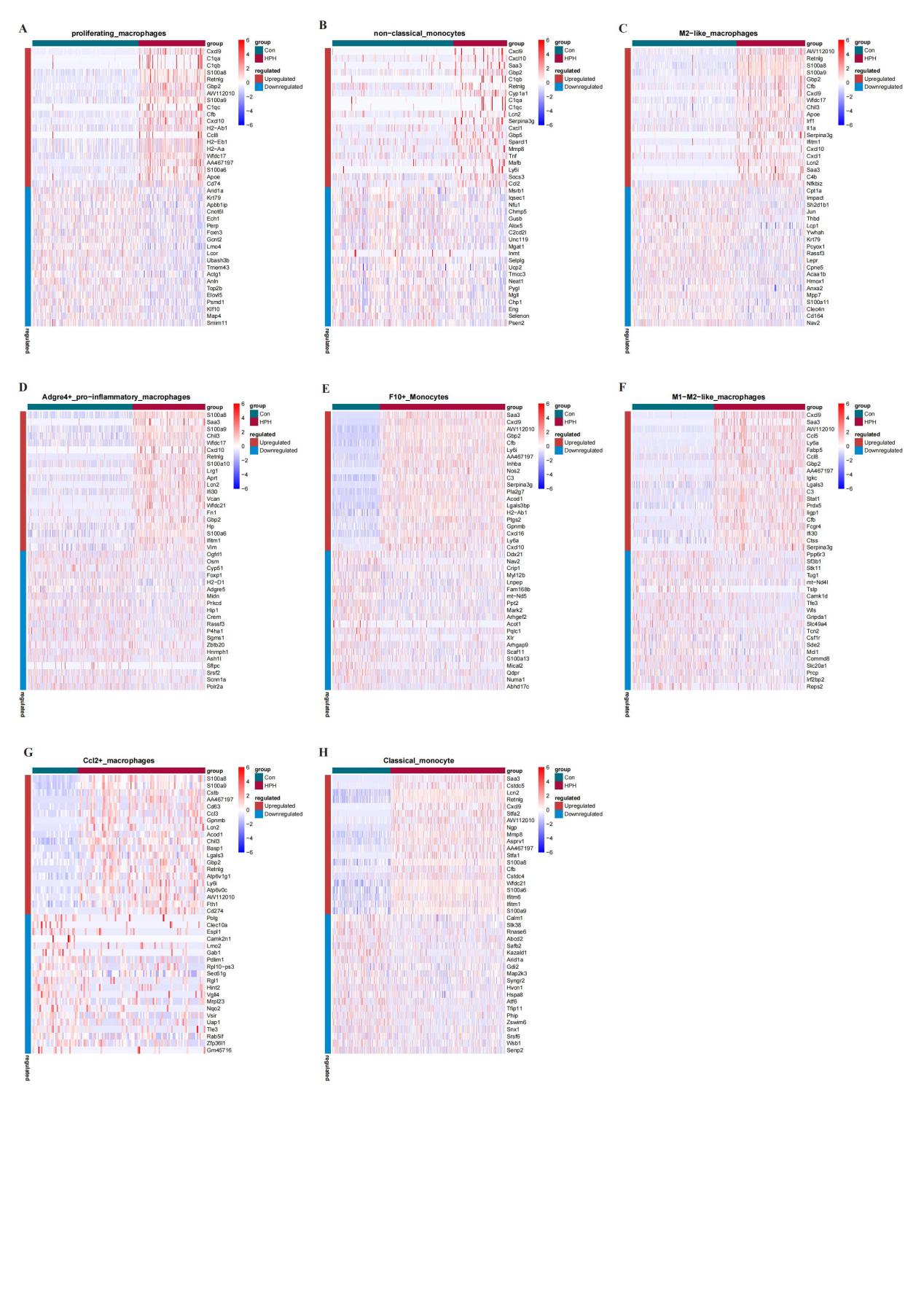
**

**Supplemental figure 15. Volcano plots showing the genes differentially expressed in different cells. (A)** Proliferating macrophages, **(B)** non-classical monocytes, **(C)** M2-like macrophages, **(D)** Adgre4^+^ pro-inflammatory macrophages, **(E)** F10^+^ monocytes, **(F)** M1-M2 like macrophages, **(G)** Ccl2+ macrophages and **(H)** classical monocytes.

**Supplemental figure 16**

**
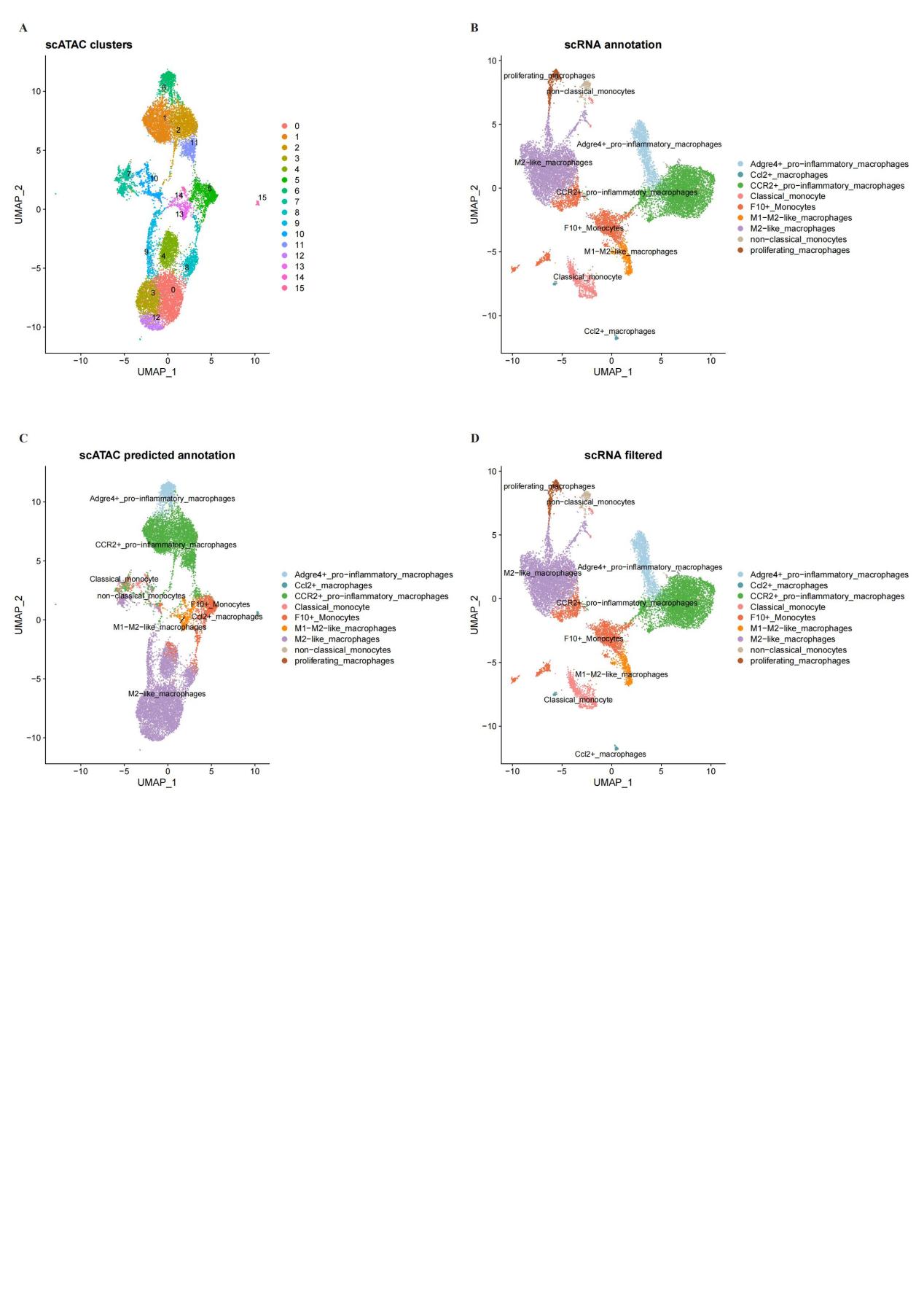
**

**Supplemental figure 16. Integration of scRNA-Seq and scATAC-Seq Datasets in macrophages. (A)** UMAP representation of scATAC-seq datasets in macrophages. **(B)** UMAP representation of scRNA-seq datasets in macrophages. **(C)** UMAP representation of scATAC predicted annotation in macrophages. **(D)** UMAP representation of scRNA filtered datasets in macrophages.

**Supplemental figure 17**


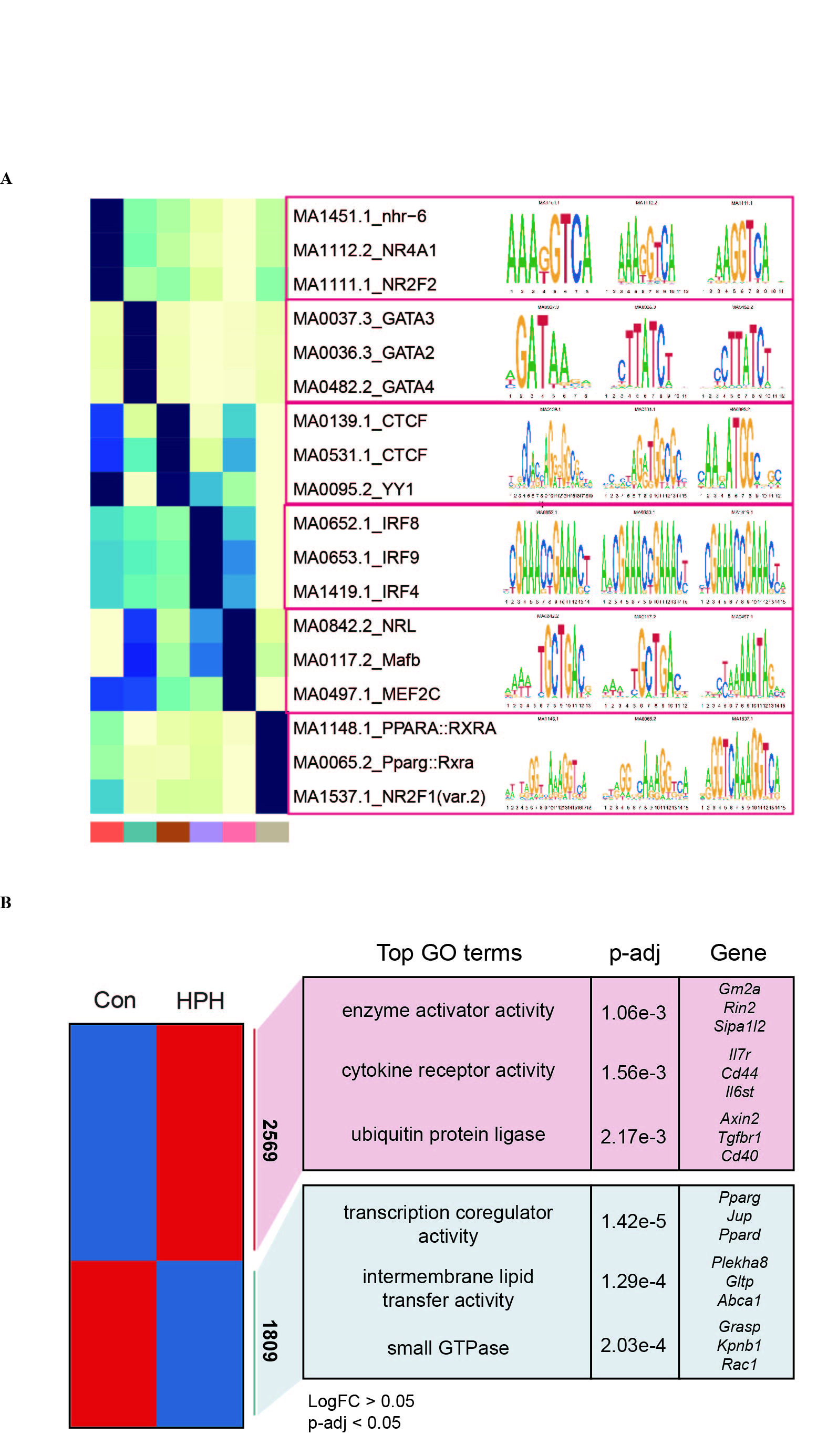


**Supplemental figure 17.** **(A)** Transcription factor heatmap of the top 3 transcription factors corresponding to each cell type and their annotated motifs (right). **(B)** GO pathways and their top genes in lung tissue from the Con and HPH groups.

**Supplemental figure 18**

**
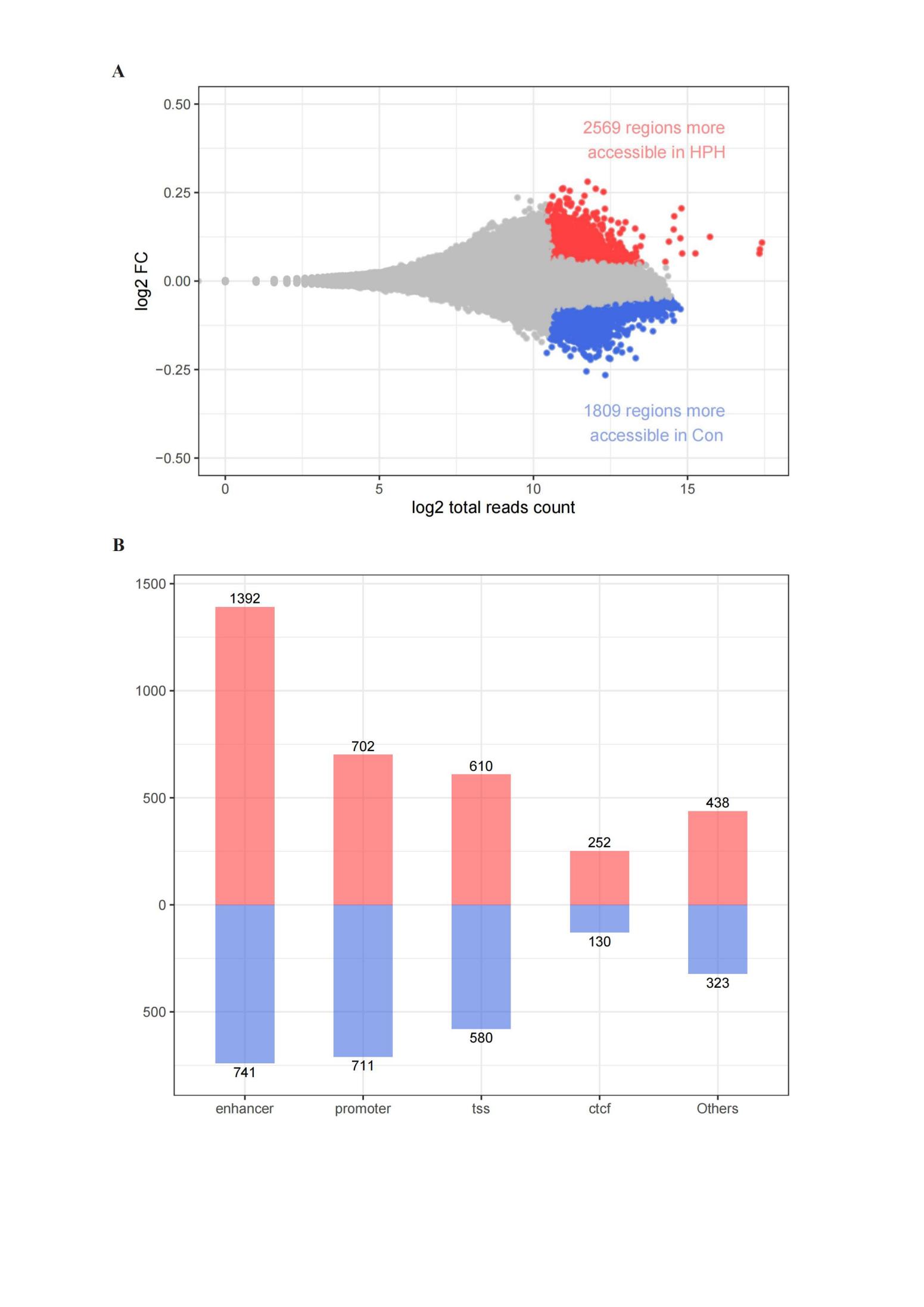
**

**Supplemental figure 18. (A)** MA plot showed the differential open regions of macrophages in the HPH (red) and Con (blue) group, and the specified number of areas was calculated. **(B)** The histograms show the number of open or closed regions at the promoter and enhancer of macrophages in the mouse lung tissue.

**Supplemental figure 19**

**
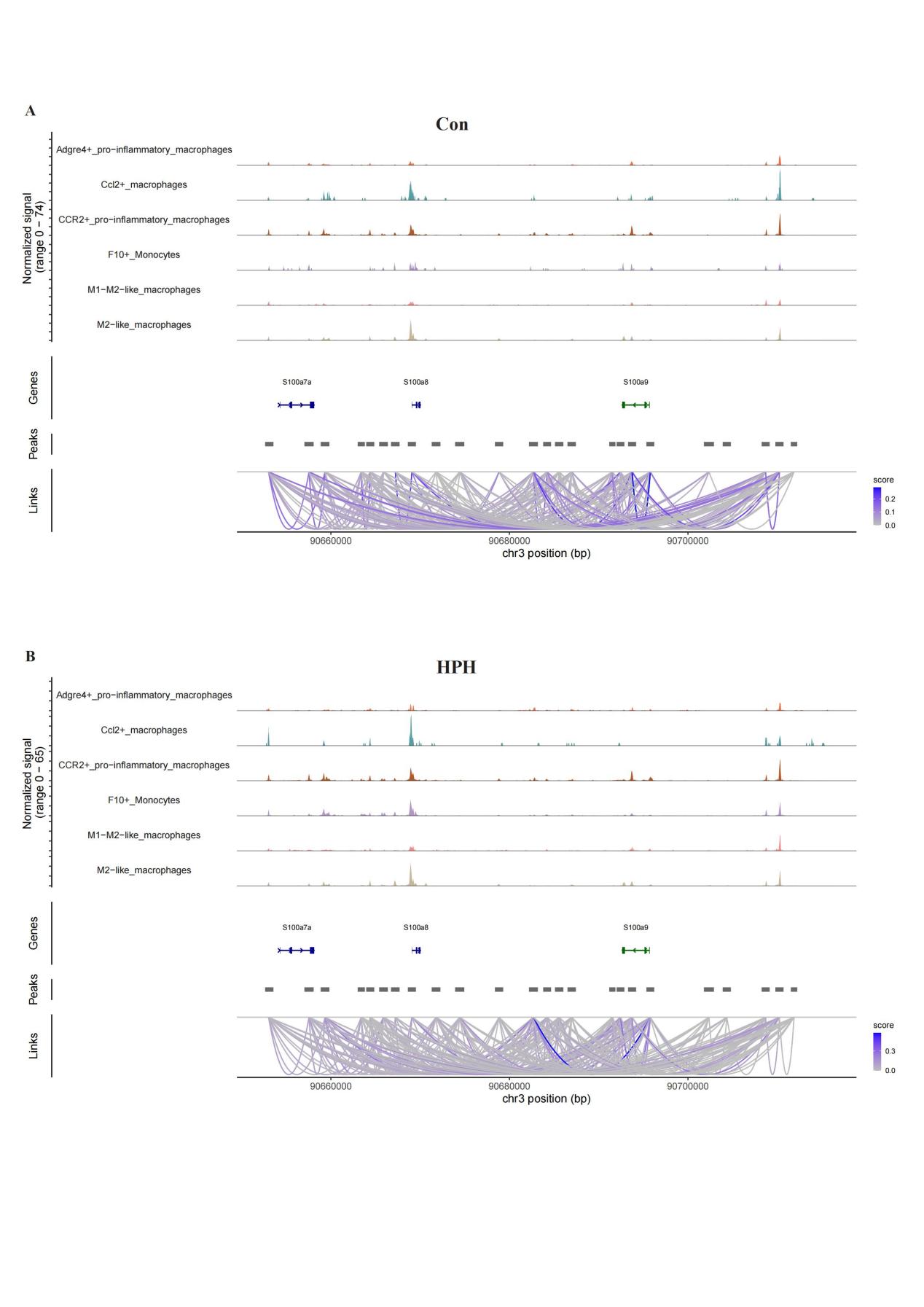
**

**Supplemental figure 19. scATAC-seq coaccessibility analysis of S100a8 and S100a9 in Con group (A) and HPH group (B) in different subtypes of macrophages.**

**Supplemental figure 20**

**
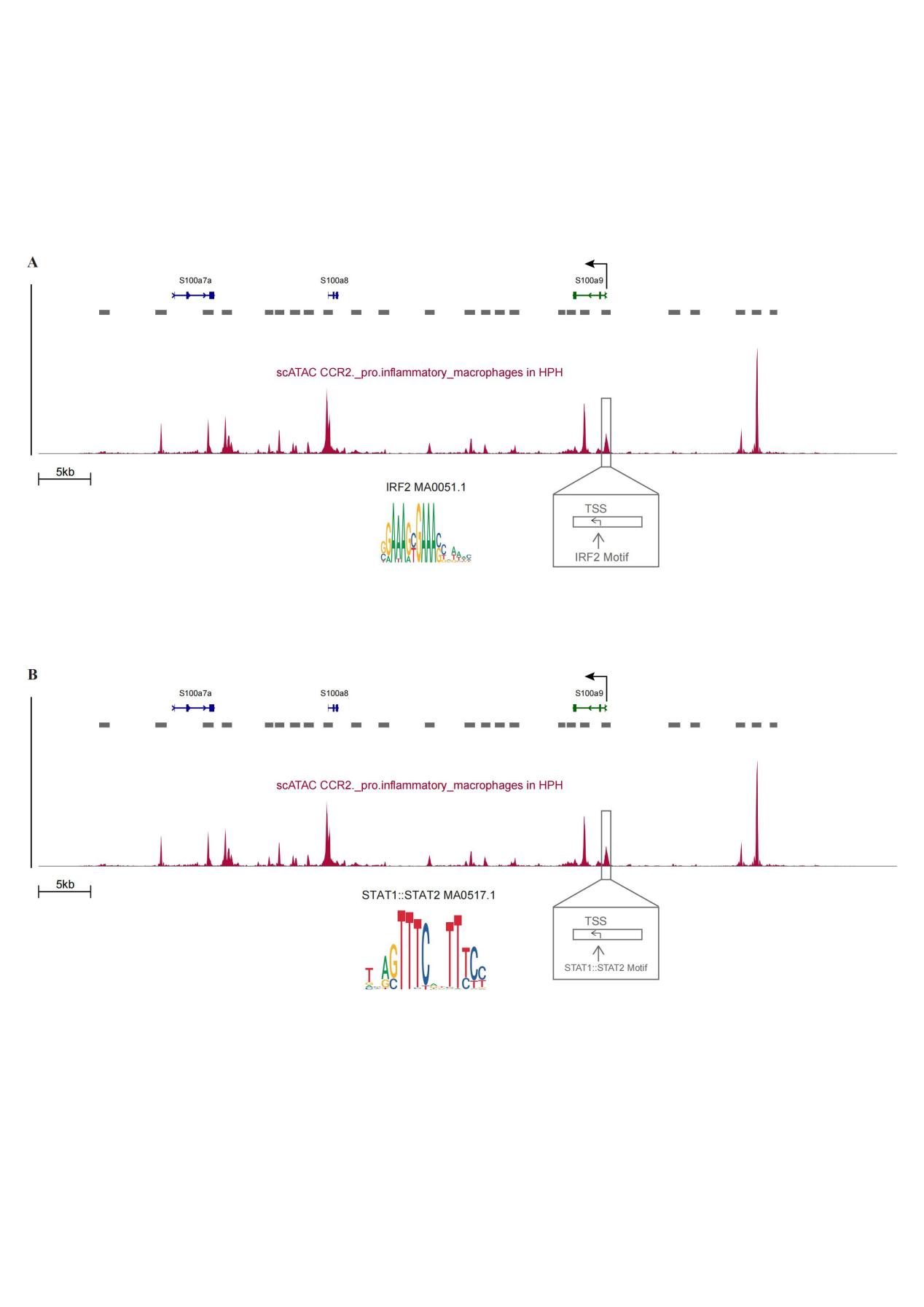
**

**Supplemental figure 20. Schematic representation of the S100a9 site with an average scATAC-seq signal in CCR2^+^ pro-inflammatory macrophage cells in HPH. (A)** IRF2 motif (MA0051.1) in the S100a9 promoter (TSS). **(B)** STAT1::STAT2 motif (MA0517.1) in the S100a9 promoter (TSS).
